# Influence of SPION Surface Coating on Magnetic Properties and Theranostic Profile

**DOI:** 10.3390/molecules29081824

**Published:** 2024-04-17

**Authors:** Vital Cruvinel Ferreira-Filho, Beatriz Morais, Bruno J. C. Vieira, João Carlos Waerenborgh, Maria João Carmezim, Csilla Noémi Tóth, Sandra Même, Sara Lacerda, Daniel Jaque, Célia T. Sousa, Maria Paula Cabral Campello, Laura C. J. Pereira

**Affiliations:** 1 Centro de Ciências e Tecnologias Nucleares, Departamento Engenharia Ciências Nucleares, Instituto Superior Técnico, Universidade de Lisboa, EN10, km 139,7, 2695-066 Bobadela, Portugal; vital.filho@ctn.tecnico.ulisboa.pt (V.C.F.-F.); beatriz.morais@ctn.tecnico.ulisboa (B.M.); brunovieira@ctn.tecnico.ulisboa.pt (B.J.C.V.); jcarlos@ctn.tecnico.ulisboa.pt (J.C.W.); 2 Centro de Química Estrutural-CQE, DEQ, Instituto Superior Técnico, Universidade de Lisboa, Av. Rovisco Pais, 1049-001 Lisbon, Portugal; maria.carmezim@estsetubal.ips.pt; 3 ESTSetúbal, CDP2T, Instituto Politécnico de Setúbal, 2910-761 Setúbal, Portugal; 4 Centre de Biophysique Moléculaire, CNRS, UPR 4301, Université d’Orléans, Rue Charles Sadron, 45071 Orléans, CEDEX 2, France; csilla-noemi.garda-toth@cnrs-orleans.fr (C.N.T.); sandra.meme@cnrs-orleans.fr (S.M.); sara.lacerda@cnrs-orleans.fr (S.L.); 5 Departamento de Física de Materiales, Universidad Autonoma de Madrid, Avda. Francisco Tomás y Valiente 7, 28049 Madrid, Spain; daniel.jaque@uam.es; 6 Departamento de Física Aplicada, Universidad Autonoma de Madrid, Avda. Francisco Tomás y Valiente 7, 28049 Madrid, Spain; celia.tsousa@uam.es

**Keywords:** superparamagnetism, iron oxides nanoparticles, gold nanoparticles, gadolinium, hyperthermia, magnetic resonance imaging

## Abstract

This study aimed to develop multifunctional nanoplatforms for both cancer imaging and therapy using superparamagnetic iron oxide nanoparticles (SPIONs). Two distinct synthetic methods, reduction–precipitation (M_R/P_) and co-precipitation at controlled pH (M_pH_), were explored, including the assessment of the coating’s influence, namely dextran and gold, on their magnetic properties. These SPIONs were further functionalized with gadolinium to act as dual T1/T2 contrast agents for magnetic resonance imaging (MRI). Parameters such as size, stability, morphology, and magnetic behavior were evaluated by a detailed characterization analysis. To assess their efficacy in imaging and therapy, relaxivity and hyperthermia experiments were performed, respectively. The results revealed that both synthetic methods lead to SPIONs with similar average size, 9 nm. Mössbauer spectroscopy indicated that samples obtained from M_R/P_ consist of approximately 11–13% of Fe present in magnetite, while samples obtained from M_pH_ have higher contents of 33–45%. Despite coating and functionalization, all samples exhibited superparamagnetic behavior at room temperature. Hyperthermia experiments showed increased SAR values with higher magnetic field intensity and frequency. Moreover, the relaxivity studies suggested potential dual T1/T2 contrast agent capabilities for the coated SP_pH_-Dx-Au-Gd sample, thus demonstrating its potential in cancer diagnosis.

## 1. Introduction

Cancer is currently the second leading cause of death, only behind cardiovascular disease [1,2]. Treatments such as chemotherapy, radiotherapy, and surgical removal of tumors are the most frequent approaches. However, patients’ survival mainly depends on an accurate and early diagnosis of the disease [2]. Inorganic nanoparticles (NPs) have been widely studied in biomedicine because of their attractive properties. These include non-toxicity, size similar to biomolecules, relative ease of preparation and functionalization with various imaging agents, biological targeting moieties and drugs. When combining therapeutic and diagnostic functions in the same tool, we have the so-called theranostic nanoparticles. Although this concept is promising, NPs need to overcome several hurdles to reach clinical applications [3,4]. Currently, most of the iron oxide drugs approved in the 1990s as clinical MRI contrast agents have been withdrawn from the market. Nanotherm^®^ is the only iron oxide nanoparticle drug to have been clinically approved since 2010 for the treatment of magnetic hyperthermia glioblastoma [5,6,7].

The development and success of targeted delivery nanoplatforms depend on the knowledge of the mechanisms and interactions of NPs with the tumor, as their pharmacokinetics and pharmacodynamics are strongly dependent on their physical and chemical properties and surface composition. Among the myriad of possible nanoparticles for biomedical applications, magnetic nanoparticles are most suitable for theranostic applications in cancer. It has been settled that these materials can be influenced by an external magnetic field, so it is clear why they are so interesting for biomedical applications. Below a certain diameter, magnetic nanoparticles behave as a single domain, a single magnetic spin, and are currently classified as a superparamagnetic material [8]. Superparamagnetism is the main physical property of the superparamagnetic iron oxide nanoparticles (SPIONs) and plays an important role in cell separation, drug and gene delivery, immunoassay, magnetic resonance imaging (MRI), surgery, magnetic hyperthermia treatment (MHT), and other therapies [8,9,10]. This behavior presents a moderately strong magnetization in the presence of an externally applied field, which disappears as the field is removed, leaving no trace of magnetization in the particles. Nonetheless, SPIONs have some shortcomings, mainly their tendency to oxidize and aggregate in vivo and their high uptake by macrophages. Thus, such nanoparticles typically require some form of functionalization to increase biocompatibility, decrease toxicity, and improve their properties, such as stability and dispersity [8,11,12]. A current strategy relies on the use of biocompatible coating agents containing an organic and/or inorganic material to improve NPs stability and make them more versatile [12,13]. A dextran vs. polyethylene glycol (PEG) coating study has been reported, highlighting the effect of the nature of the organic coating polymer on the magnetization performance of SPIONs. Indeed, dextran-based polymer with short chains does not affect the magnetization performance of SPIONs, while longer-chain polymer-based PEG leads to a substantial magnetization decrease [14]. Thanks to their high versatility, gold-coated magnetic nanoparticles can be used for many applications. Their optical and magnetic properties can be tuned and tailored by changing their size, gold shell thickness, shape, charge, and surface modification by the conjugation of various biomolecules such as peptides, antibodies, aptamers, and (radio) metals. Nevertheless, depending on the gold shell thickness, charge, and surface modification, the gold coating can dramatically affect the magnetic properties of the final nanoparticles, ultimately affecting their biomedical performance [15].

We have previously reported on gold nanoparticles (AuNPs) functionalized with a macrocyclic thiol-DO3A derivative and with a bombesin peptide analog, which have shown a favorable affinity towards gastrin-releasing peptide receptor (GRPR), remarkably high internalization into human pancreatic PC3 cancer cells, T1/T2 MRI properties and promising properties as radiosensitizers [16,17,18,19].

Motivated by the above-reported results, we decided to evaluate and compare multifunctional nanoplatforms based on superparamagnetic iron oxide nanoparticles (Naked SPIONs) synthesized by two different chemical methods: reduction–precipitation (M_R/P_) and co-precipitation at controlled pH (M_pH_) of the iron precursor. The obtained naked SPIONs were protected with a dextran-based polymer, coated with a gold shell, and functionalized with gadolinium to act as dual T1/T2 contrast agents for MRI.

This study aims to improve the understanding of the parameters that influence good magnetic performance and, consequently, the imaging and therapeutic properties of SPIONs. All synthesized nanomaterials were characterized in terms of size, stability, and morphology using a combination of techniques such as UV-Vis, PXRD, FTIR, TEM, DLS, and Zeta Potential. A thorough and systematic magnetic study by Mössbauer spectroscopy and by static magnetization allowed us to assess their magnetic performance, namely in terms of superparamagnetic (single domain) and blocking behaviors. Once the magnetic properties have been determined, the most promising nanostructures were selected for assessment of their potential use as dual T1/T2 MRI contrast agents and for hyperthermia to assess their theranostic behavior.

## 2. Results and Discussion

**Scheme 1:**
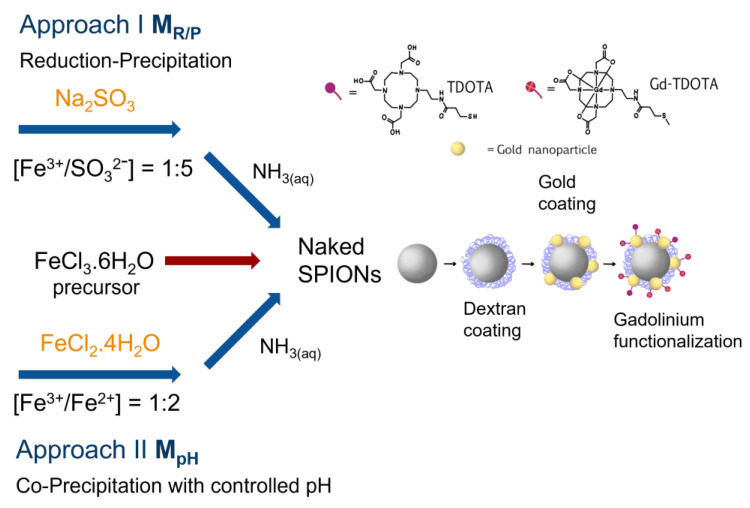
Steps in the synthesis of SPIONs obtained by the reduction-precipitation process (M_R/P_) and the pH-controlled Co-Precipitation Process (M_pH_).

In this work, SPIONs were synthesized by two different methodologies based on previously described procedures reduction–precipitation [14] and controlled co-precipitation (NH_3_ aqua, pH 9.6) of iron salts [20] (Scheme 1). To evaluate the effect of the type of stabilizing coating used to protect them, the obtained SPIONs were coated with a dextran polymer and with gold and functionalized with gadolinium. Table 1 summarizes the denomination of each SPION sample according to the methods obtained.

In the reduction–precipitation process, Fe^3+^ is initially converted to Fe^2+^ using sodium sulfite, and subsequently, Fe_3_O_4_ is precipitated via titration with NH_3_. The physicochemical characteristics of the resulting SPIONs in this procedure are partially determined by the [Fe^3+^/SO_3_^2−^] molar ratio applied. In the aforementioned synthesis, a molar ratio of [Fe^3+^/SO_3_^2−^] 1/5 was employed:

$${FeCl}_{3}\cdot 6H_{2}O+5{Na}_{2}SO_{3}\to Fe{Cl}_{2}+2NaCl+3{Na}_{2}SO_{4}+9H_{2}O$$

$${Fe}^{3+}+{Fe}^{2+}+8OH^{-}\to {Fe}_{3}O_{4}+4H_{2}O$$

In the co-precipitation process with controlled pH, ferrous chloride (FeCl_2_) and ferric chloride (FeCl_3_) are dissolved separately in water to create two precursor solutions. These solutions are then combined in a 2:1 molar ratio, ensuring an excess of Fe^2+^ ions relative to Fe^3+^. While the majority of similar reactions typically employ a 1:2 molar ratio, we opted to follow the procedure outlined by Saraiva et al. [20], who obtained SPIONs using a molar ratio of 2:1, resulting in nanoparticles with very interesting characteristics. Ammonia (NH_3_) is gradually added to the resulting solution with continuous stirring until it reaches pH = 9.6. It is anticipated that NH_3_ will react with the Fe^2+^ and Fe^3+^ ions, forming the intermediate iron hydroxide (Fe(OH)_2_). Subsequently, upon reacting with the oxygen available in the reaction environment, Fe(OH)_2_ undergoes oxidation, leading to the formation of magnetite nanoparticles (Fe_3_O_4_):

$$2{FeCl}_{3}\cdot 6H_{2}O+4{FeCl}_{2}\cdot 4H_{2}O+12NH_{4}OH\to 6Fe(OH{)}_{2}+12NH_{4}Cl+28H_{2}O+Cl_{2}↑$$

$$6Fe(OH{)}_{2}+O_{2}\to 2Fe_{3}O_{4}+6H_{2}O$$

### 2.1. UV-Vis and ATR-FTIR

As already mentioned, coatings must strike a balance between providing stability and maintaining the superparamagnetic properties of SPIONs, allowing them to respond to external magnetic fields. Dextran is a natural and biodegradable polysaccharide with high biocompatibility, which reduces the risk of adverse immune responses or toxicity when used in vivo. Dextran coatings can improve the colloidal stability of SPIONs under physiological conditions, preventing rapid elimination by the immune system and prolonging their circulation time in the bloodstream. Although these advantages make dextran-coated SPIONs a promising nanomaterial for various in vivo applications, it is important to note that these polymeric coatings can also modify, among other parameters, the size and surface charge of the particles, which are key factors to maximize the potential of coated SPIONs in specific applications [14,21].

**Figure 1:**
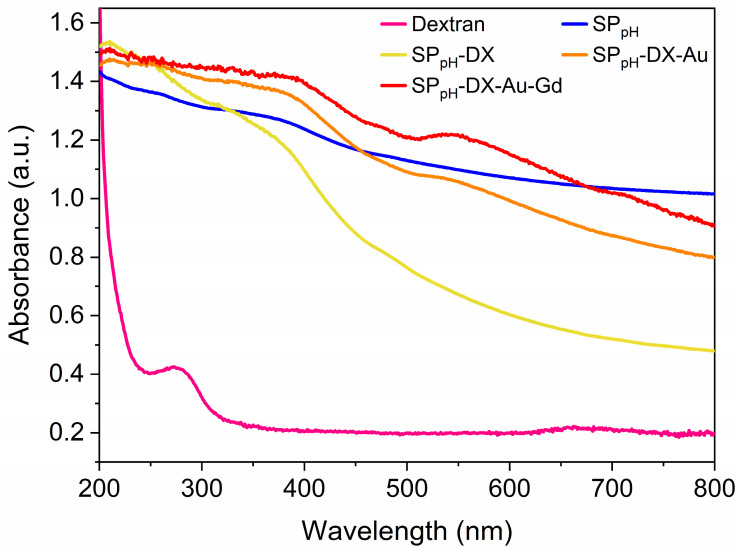
UV-Vis spectra of Dextran (pink) and M_pH_ samples, SP_pH_ (blue), SP_pH_-Dx (yellow), SP_pH_-Dx-Au (orange), and SP_pH_-Dx-Au-Gd (red).

Metal nanoparticles interact strongly with specific wavelengths of light, exhibiting strong dipolar excitations in the form of localized surface plasmon resonances (LSPR). Small changes that occur in the molecular layer of the metal surface usually result in dramatic changes in the position and pattern of the LSPR. Accordingly, SPR is a powerful tool for evaluating the success of the synthesis and post-synthesis of successive functionalization of SPIONs. Therefore, the formation of the SPIONs and the presence of the different conjugated coatings were first confirmed by UV-Vis spectroscopy. As an example, Figure 1 shows the UV-Vis absorbance spectra of the bare SPIONs obtained by the co-precipitation method and of the corresponding SPIONs with successive coatings. The UV-Vis spectrum of the naked SP_pH_ shows two large and broad absorption peaks that are positioned in the region of 220–270 nm and 310–390 nm, characteristic of iron oxide nanoparticles, namely magnetite nanoparticles [21,22,23,24,25,26]. The pattern of the SP_pH_-Dx spectrum is quite different from that of its precursor SP_pH_, with a noticeable decrease in absorption with the increasing of the wavelength light (200 to 800 nm). The broad peak around 390 nm indicates that the SPIONs are surrounded by the dextran polymer. The more pronounced SPR bands observed in the spectra of SP_pH_-Dx-Au and SP_pH_-Dx-Au-Gd, with maxima in the 520–530 nm regions, clearly indicate the presence of Au on the surface of the respective SPIONs [16,17].

The pattern of the SP_pH_-based SPIONs’ spectra is similar to those of the respective ones obtained by the reduction–precipitation method (Figure S1). The absorption maxima observed for each nanostructure are listed in Table S1 of the supplementary material.

**Figure 2:**
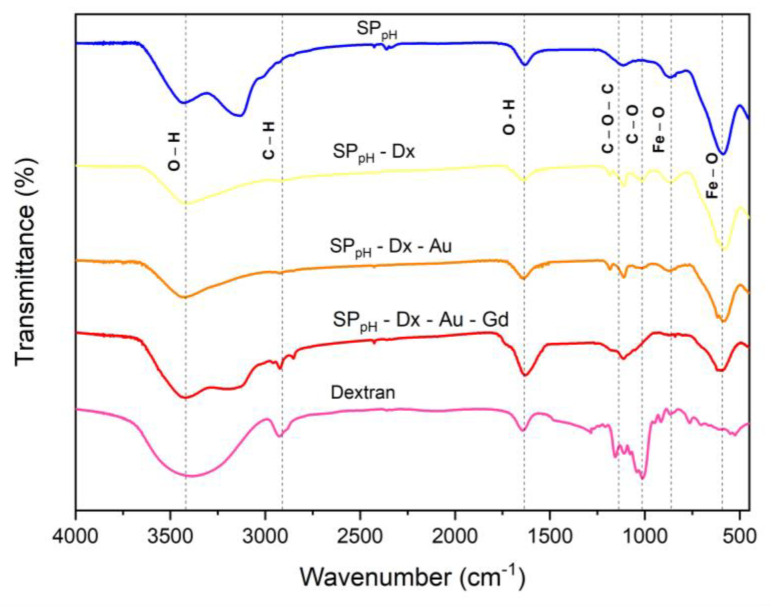
ATR-FTIR spectra of Dextran (pink) and M_pH_ samples, SP_pH_ (blue), SP_pH_-Dx (yellow), SP_pH_-Dx-Au (orange), and SP_pH_-Dx-Au-Gd (red).

The ATR-FTIR spectra are consistent with the coating of SPIONs with Dextran (see Figure 2 and Table S2). The spectra of the naked SP_R/P_ and SP_pH_ show a broad band at about 3400 cm^−1^, characteristic of the stretching vibrations of –OH groups in water molecules and hydroxyl groups present on the surface of the SPIONs. The band near 1640 cm^−1^ is also associated with bending vibrations of H_2_O adsorbed on the surface of the nanoparticles. The bands observed at low frequency (1000–500 cm^−1^) are due to the iron oxide matrix: the weak band around 870 cm^−1^ is related to the stretching vibrations of the Fe–O excited bonds from the γ-Fe_2_O_3_ [27,28], the pattern of the strong band around 580 cm^−1^ is related to the bending vibrations of Fe–O in magnetite (Fe_3_O_4_) and the broad band observed around 620 cm^−1^ is consistent with the presence of maghemite (γ-Fe_2_O_3_) [27,28].

The FTIR spectrum of dextran reveals its structure, which arises from the repeated connection of glucose molecules through glycosidic bonds, forming chain-like structures characteristic of polysaccharides. The strong band centered at 3400 cm^−1^ is representative of the OH bonds, the peaks around 2920 cm^−1^ and around 2850 cm^−1^ are due to the asymmetric and symmetric stretching vibration of the CH_2_ groups of the Dextran polymer, respectively, and the band located at 1643 cm^−1^ is assigned to the C–C (in-plane of the ring). The band at 1157 cm^−1^ is probably related to the C–O stretching vibration of the glycosidic bonds, and the strong and sharp band at 1013 cm^−1^ is attributed to the C–O stretching vibration of the hydroxyl groups in the Dextran polymer [29]. In the spectra of SPIONs coated with Dextran (SP_R/P_-Dx and SP_pH_-DX), distinctive absorption peaks emerge, indicating the successful coating of dextran onto the SPION surface. However, upon coating these SPIONs with gold (SP_R/P_-Dx-Au; SP_pH_-DX-Au, and SP_R/P_-DX-Au-Gd; SP_pH_-DX-Au-Gd), the intensity of the bands attributable to dextran noticeably decreases, as expected.

### 2.2. Powder X-ray Diffraction (PXRD)

**Figure 3:**
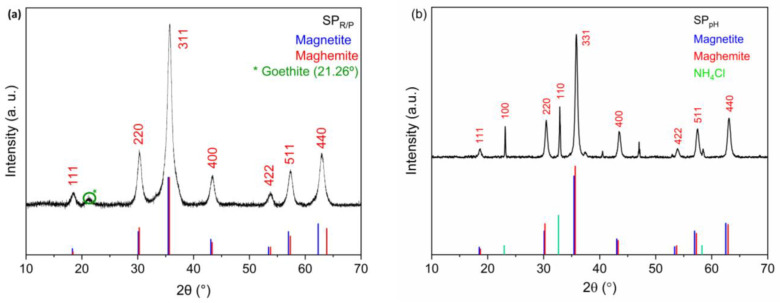
Powder XRD diffractograms of samples (**a**) SP_R/P_ and (**b**) SP_pH_.

Powder X-ray diffraction (PXRD) analysis was performed to obtain information on the structural nature, phase, and approximate crystalline core size of the SPIONs, allowing the identification of the main peaks of a spinel phase (Figure 3). Unit-cell parameters estimated from the PowderCell program are between those of magnetite with ideal stoichiometry Fe_3_O_4_ (JCPDS file 19-629) and maghemite γ-Fe_2_O_3_ (JCPDS file 39-1346). Fe_3_O_4_ and γ-Fe_2_O_3_ are isostructural, having diffraction peaks at approximately the same diffraction angles. This fact makes it difficult to distinguish between magnetite and maghemite with this technique. The diffraction pattern showed peaks at 2θ of approximately 18.50°, 30.31°, 35.72°, 43.40°, 53.80°, 57.35°, and 62.94°, allowing the identification of the main peaks of a spinel phase. These peaks correspond to the crystallographic planes (111), (220), (311), (400), (422), (511), and (440). Figure 3 shows the diffractograms of SPIONs, SP_R/P_, and SP_pH_.

As shown below, Mössbauer spectroscopy revealed that magnetite in the samples is partially oxidized. There likely is no uniform level of oxidation across all the studied spinel nanoparticles. These particles probably range in oxidation state, with some being as oxidized as maghemite, where all the iron is Fe^3+^. This variation in oxidation level creates a mix of nanodomains with different unit-cell parameters within the samples, which contributes to broadening the X-ray diffraction peaks along with the effect of the small grain sizes. The diffractogram of SP_R/P_ shows a peak at 21.26° that reveals the presence of a small amount of goethite [30]. The diffractogram of the naked SPIONs, SP_pH_, indicates the presence of an ammonium chloride salt phase (NH_4_Cl). The main peaks of this salt represented in the diffractogram are 23.05°, 32.76°, 40.39°, 46.94°, 52.86°, and 58.31°, which agree with those reported in the literature (JCPDS file 73-1491) [31].

**Figure 4:**
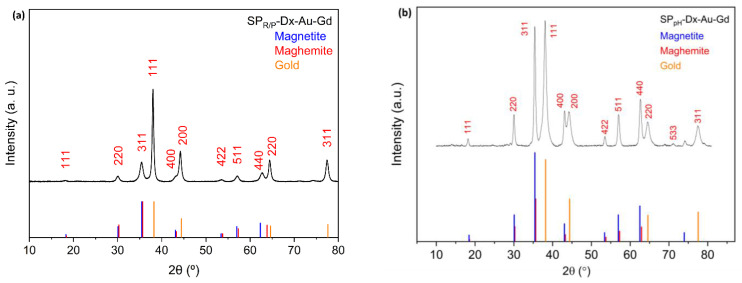
Powder XRD diffractograms of samples (**a**) SP_R/P_-Dx-Au-Gd and (**b**) SP_pH_-Dx-Au-Gd.

Figures S5 and S6 show the powder X-ray diffractogram of the coated SP_R/P_ (samples SP_R/P_-Dx, SP_R/P_-Dx-Au) and SP_pH_ (samples SP_pH_-Dx, SP_pH_-Dx-Au), respectively. The X-ray diffraction measurements for SP_R/P_-Dx-Au-Gd, and SP_pH_-Dx-Au-Gd, displayed peaks at 2θ for the gold phase at, approximately, 38.15°, 44.32°, 64.56°, 77.53°. Compared with the JCPDS card for gold (Au^0^) (JCPDS 04-0784), these peaks correspond to the crystallographic planes (111), (200), (220), and (311) of the face-centered cubic structure. Figure 4 shows the diffractograms of SP_R/P_-Dx-Au-Gd and SP_pH_-Dx-Au-Gd nanoparticles. Even though the samples have a higher percentage of iron than gold, the peaks for gold are more intense due to the higher scattering factor of the gold nanoparticles [32], causing the iron oxide peaks to be somewhat faded. Using the three main peaks from PXRD data of the samples, the crystallite size (DXRD) of the iron core of each sample was estimated using Debye Scherrer’s equation (see Section 3.4.3) [25]. The values are summarized in Table 2. The closeness of DXRD values for both types of naked SPIONs, SP_R/P_ and SP_pH_: 9.04 ± 0.6 nm and 9.98 ± 0.5 nm, respectively, indicates that the two different synthetic routes lead to similar magnetite crystalline sizes. These values are also in agreement with those usually reported for naked SPIONs [33,34]. For the dextran-coated SPIONs, a wider range of values was obtained: from 7.38 ± 0.9 nm for SP_R/P_-Dx to 12.04 ± 0.6 nm for SP_pH_-Dx. At first consideration, this fact may be only due to differences in the synthetic route and coating. However, it could also be related to the crystal shape of the nanoparticles. Indeed, the variable K in Scherrer’s equation is associated with the crystal shape, and the value of K = 0.9 is commonly accepted, although it can vary between 0.62 and 2.08. In fact, this definition is only strictly adequate when the size and shape of the crystals are fairly uniform. These values are consistent with those found in other SPIONs coated with dextran-based polymers [14,20,21,32,35,36,37,38,39,40,41].

### 2.3. Transmission Electron Microscopy (TEM) and Dynamic Light Scattering (DLS)

The transmission electron microscopy (TEM) technique was used to evaluate the size, shape, and size dispersion (σ) of the different produced SPIONs (Equation (2)). The average nanoparticle size (D_TEM_) was calculated from the TEM images using the ImageJ program (version 1.35J).

The obtained values are shown in Table 2 and corroborate the calculations performed previously using PXRD, indicating that the nanoparticles are mainly single crystallites [42]. They are also in line with those reported in the literature for this type of nanoparticles [39,40,43,44,45]. From the obtained values, the histograms presented were constructed using sample sizes (N) of 100 and 200. 

**Figure 5:**
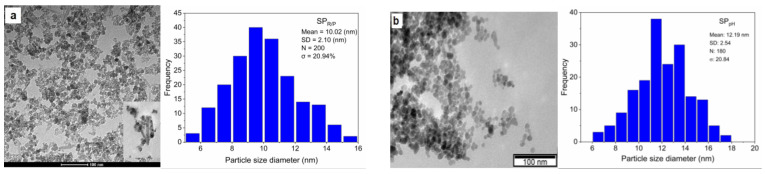

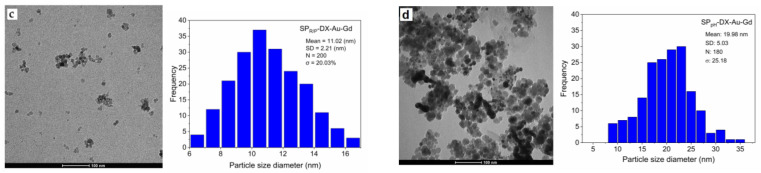
Transmission electron microscopy images of the SPIONs and respective size histogram: (**a**) SP_R/P_; (**b**) SP_pH_; (**c**) SP_R/P_-Dx-Au-Gd; (**d**) SP_pH_-Dx-Au-Gd.

The SP_R/P_ nanoparticles (Figure 5a and Figure S7) are relatively well dispersed, with a D_TEM_ of 10.0 ± 2.1 nm. A mixture of spherical, cubic, and some undefined shapes can be observed, with a σ of 20.9% (Figure 5a, Table 2). As expected, D_TEM_ values are slightly higher than DXRD since the former is related to the particle size and the latter only to its crystallite. As evidenced by the PXRD technique, the SP_R/P_ sample presents a peak related to goethite, an iron oxide–hydroxide. Through TEM images, it was possible to confirm its presence, as shown by the elongated crystals next to the nanoparticles (Figure 5a, inset). If the presence of goethite is predominant in the sample, it may affect the efficacy of these nanoparticles for biomedical applications since its shape is not favorable, and the hydroxide groups are not biocompatible. In addition, goethite has a lower magnetic moment compared with magnetite or maghemite. The TEM images for the SP_pH_ SPIONs (Figure 5b and Figure S8) showed good results with a size dispersion of 20.8%, a well-defined spherical shape with a few variations, and a D_TEM_ of 12.2 ± 2.5 nm. In addition, they are better dispersed than SP_R/P_. Larger nanoparticles are found to be close but not aggregated, which may result from long-range magnetic dipole–dipole interaction between the nanoparticles [42]. Coating the naked SP_R/P_ with dextran and gold, SP_R/P_-Dx-Au (Figure S7) produced nanoparticles with D_TEM_ = 10.9 ± 2.7 nm and σ = 24.84%.

Functionalization of these SPIONs with gadolinium, SP_R/P_-Dx-Au-Gd (Figure 5c), had no noticeable influence on their size, with a D_TEM_ = 11.0 ± 2.8 nm, σ = 24.2%. In contrast, the successive coating of SP_pH_ SPIONs consistently resulted in an increase in their size. Namely, there was an increase of more than 50% in size for SP_pH_-Dx-Au-Gd (Figure 5d) compared with SP_pH_ (20.0 ± 5.0 nm vs. 12.2 ± 2.5 nm, respectively). It is, however, noteworthy that the nanoparticles are moderately dispersed, with some areas of higher nanoparticle concentration.

The Dynamic Light Scattering (DLS) analyses of the SPIONs showed different hydrodynamic sizes, comprehended between 25 nm and 352 nm. As can be inferred from Table 2, the hydrodynamic size of the SPIONs is very sensitive to changes in the surface coating and functionalization with gadolinium. The observed increase in hydrodynamic size from the naked SPIONs and the coated SPIONs with Dextran is expected. Dextran is a hydrophilic polymer that forms a protective layer around the nanoparticles. The hydrodynamic diameter of SP_R/P_-Dx is about 6-fold higher than that of the SP_R/P_, and the one of SP_pH_-Dx is about 5-fold higher than SP_pH_. A significant reduction in hydrodynamic size is observed after gold coating, indicating the formation of a thin, relatively closely packed gold layer on the surface of the SPIONs previously coated with Dextran. It was observed that the coatings rearrangements post stable coordination of Gd^3+^ ions led to an increase in the hydrodynamic size of the nanoparticles coated with Dextran and gold (SP_R/P_-Dx-Au-Gd and SP_pH_-Dx-Au-Gd).

The polydispersity index (PDI) observed for SPIONs shows a relatively low monodispersity distribution and is consistent with the tendency of these nanoparticles to agglomerate/aggregate in aqueous solution during the DLS measurement process [38,46]. Concerning MRI issues, polydispersity can have both advantages and disadvantages. The size of SPIONs influences their relaxivity, impacting MRI contrast. A polydisperse sample might have a broader range of relaxivities, potentially leading to improved contrast for certain imaging applications. However, it can also introduce challenges in reproducibility and control, which are essential for consistent imaging results [39,41,47,48].

The zeta potential is a measure of the electric charge on the surface of nanoparticles in a colloidal suspension. It indicates the extent of repulsion between charged particles, which affects their stability and interactions with other particles or surfaces. Thus, this parameter is a keystone factor used to predict the stability of nanoparticles in terms of monodispersity (or agglomeration) in solution. Values above ±25 mV are indicative of a high stability, whereas values between 0 mV and ±5 mV are indicative of a fast aggregation of the particles [49].

Zeta values, ζ, determined for all samples, are also presented in Table 2. Naked samples SP_R/P_ and SP_pH_ show ζ of 40.3 ± 8.3 and 43.8 ± 10.0 mV, respectively. This indicates good stability and dispersion of the particles, as also confirmed by the TEM analyses. After coating SPIONs with dextran, the zeta potential is influenced by the interaction of ions in the aqueous dispersion with the polysaccharide structure of dextran. The negative OH^−^ groups of dextran replace other adsorbed ions on the surface of the SPIONs, leading to a decrease in the zeta potential. Specifically, in SP_R/P_-Dx, the zeta potential decreases to a value of −6.2 ± 7.6 mV, while in SP_pH_-Dx, it decreases to −15.1 ± 1.3 mV. These values may indicate some instability and reduced dispersion of the suspensions. It is important to note that after coating the SPIONs with gold, the surface charge becomes more negative. This phenomenon can be attributed to the presence of gold nanoparticles surrounded by a cloud of anions, which further contributes to the negative charge on the surface [50,51]. Therefore, the zeta potential changes to −19.1 ± 6.0 mV in SP_R/P_-Dx-Au and to 21.5 ± 0.7 mV in SP_pH_-Dx-Au.

In the next step, there is a significant increase in the surface charge after gadolinium functionalization, which is mainly due to the presence of gadolinium ions (Gd^3+^). These ions confer positive surface charges to the SPIONs. Specifically, functionalization with TDOTA complexed Gd^3+^ led to an increase in ζ from −19.1 ± 6.0 mV to 35.4 ± 8.5 mV in SP_R/P_-Dx-Au-Gd and from −21.5 ± 0.7 mV to −16.7 ± 0.7 mV in SP_pH_-Dx-Au-Gd. This increase may indicate a stabilization of the nanoparticles, which is particularly evident at lower pH conditions [52]. This observed trend highlights the impact of surface modification with gadolinium on the colloidal stability of the nanoparticles, especially in acidic environments. It suggests a potential for improved stability and dispersion of the nanoparticles following gadolinium functionalization, which is crucial for cancer theranostic applications.

### 2.4. ^57^Fe Mössbauer Spectroscopy

^57^Fe Mössbauer spectroscopy measurements were carried out in order to better characterize the iron oxides in the NPs based on the iron coordination and oxidation state. The spectra of the SP_R/P_, SP_R/P_-Dx, and SP_R/P_-Dx-Au samples taken at room temperature (Figure S9), showing six broad absorption bands and a doublet, are characteristic of superparamagnetic iron oxide nanoparticles. These features appear because the NPs are tiny crystals, each containing just one magnetic domain, and their small size allows thermal energy fluctuations to rapidly flip the direction of the particle's magnetization at a rate exceeding or similar to the Larmor precession frequency of the nuclear magnetic moments in the magnetic hyperfine field [53]. If the NPs have a range of sizes, the spectra will be a combination of signals from each size, each with its own relaxation frequency.

Cooling the material to 4 K significantly slows the flipping of the magnetic moments of the NPs. This allows us to see sharper peaks in these data (Figure S9). However, a single magnetic sextet cannot explain the spectra. We need three separate sextets to fit these data accurately. Based on this analysis (Table S4), it appears that two of these sextets likely come from Fe^3+^ located on octahedral and tetrahedral sites in maghemite and magnetite [14,54], and the third sextet with the highest isomer shift suggests Fe^2+^ in magnetite below the Verwey transition [14,55]. These data allow the estimate of the Fe fraction in maghemite and magnetite domains. For instance, the analysis of the SP_R/P_ spectrum at 4 K shows that ~3.8% of Fe is present as Fe^2+^. This implies that approximately 11% of the Fe in the sample is present in magnetite nanodomains (3.8% Fe^2+^, plus 3.8% Fe^3+^ on the octahedral sites, plus 3.8% Fe^3+^ on the tetrahedral sites). For SP_R/P_-Dx and SP_R/P_-Dx-Au, approximately 11% and 13% of Fe are present in magnetite nanodomains, respectively.

The room temperature spectra of SP_R/P_, SP_R/P_-Dx, and SP_R/P_-Dx-Au may be fitted with distributions of magnetic hyperfine fields, *B_hf_*, in order to simulate the magnetic relaxation signals and a quadrupole doublet, which corresponds to the particles with the fastest magnetic relaxation [14,56]. No additional structural information may be obtained from these spectra, whose shape is mainly determined by the magnetic relaxation effects. Reasonable fits may be obtained assuming isomer shifts, IS, consistent with high-spin Fe^3+^ and Fe^2.5+^. These IS values are kept constant during the refinement procedures, which leads to results consistent with those obtained for the 4 K spectra.

**Figure 6:**
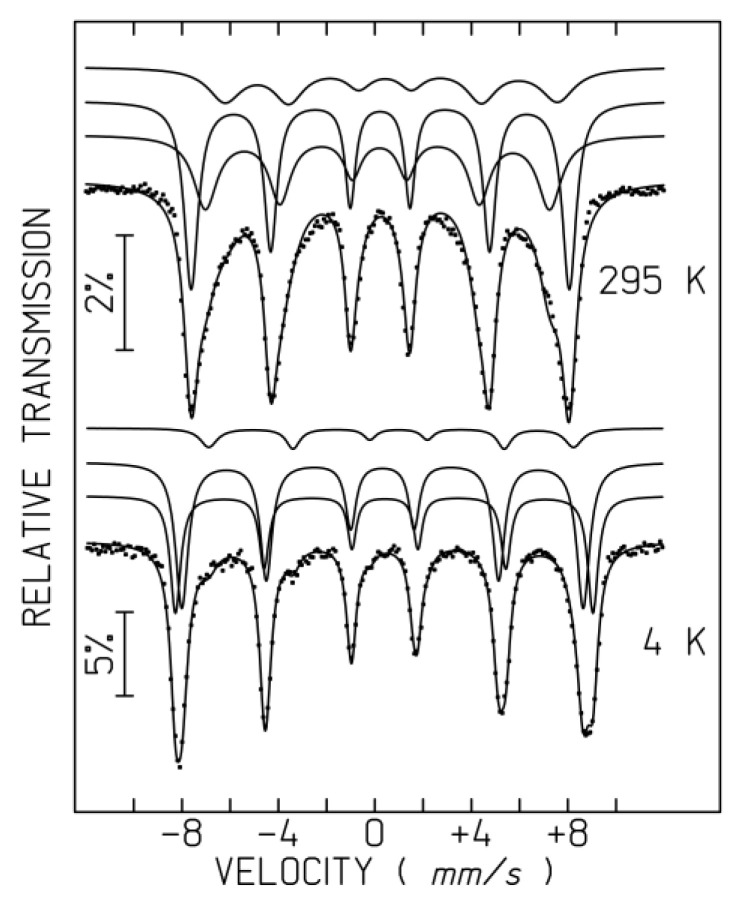
Mössbauer spectra of the SP_pH_ sample taken at 295 K and 4 K. Calculated lines on the experimental points are the sum of three sextets shown slightly shifted for clarity (Table S3).

The room temperature spectra of SP_pH_, SP_pH_-Dx, SP_pH_-Dx-Au, and SP_pH_-Dx-Au-Gd (Figure 6 and Figure S10) reveal that the magnetic relaxation of the iron oxide NPs in these samples is significantly slower than in SP_R/P_, SP_R/P_-Dx and SP_R/P_-Dx-Au [56]. The spectra of the SP_pH_ samples show no quadrupole doublet and only six asymmetrically broadened absorption peaks, but significantly less broadened than the distribution of *B_hf_* observed for the SP_R/P_ samples at 295 K. The SP_pH_ spectra at this temperature may, therefore, be properly analyzed if three magnetic splittings are considered [20]. One of the sextets is typical of the fast electron hopping observed between Fe^2+^ and Fe^3+^ on the octahedral sites of magnetite above the Verwey transition (Table S3). These Fe cations are usually reported as Fe^2.5+^ [55,57]. The remaining two sextets are consistent with Fe^3+^ on octahedral sites of maghemite and unresolved contributions of Fe^3+^ on tetrahedral sites of both magnetite and maghemite domains. Mössbauer data suggest, therefore, that approximately 33–45% of the Fe cations in the SP_pH_ samples are in magnetite domains, while the remaining Fe cations are in maghemite domains.

### 2.5. Magnetic Characterization by Magnetometry

Considering the importance of having magnetic nanoparticles with superparamagnetic behavior at room temperature to improve their potential therapeutic applications, such as hyperthermia and MRI [3,4], the magnetic characterization of all samples was performed at each step of coating procedures. This characterization used static (DC) magnetization by measuring the hysteresis loops at two different temperatures, 300 K and 10 K, and the thermal dependence of the magnetization after the zero-field-cooling (ZFC) and field-cooling (FC) cycles.

#### 2.5.1. Samples Obtained with the Reduction–Precipitation Process (SP_R/P_)

**Figure 7:**
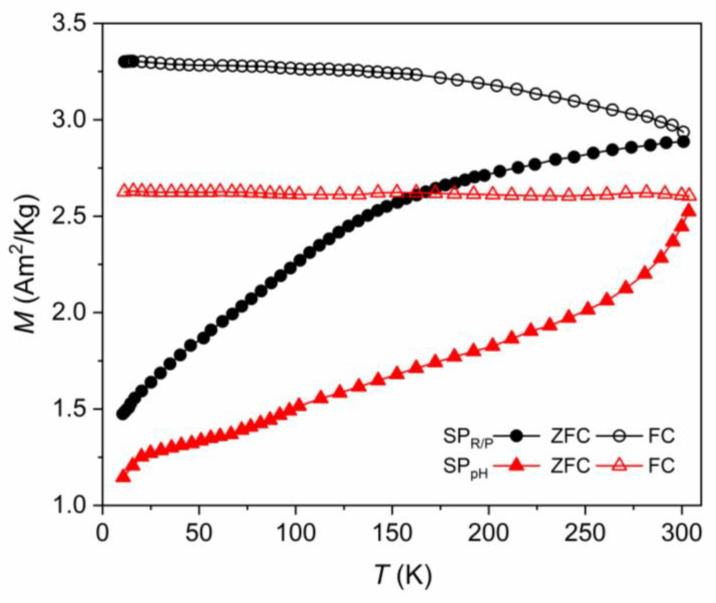
Temperature dependence of the zero-field cooling (ZFC) and field cooling (FC) magnetization for samples SP_R/P_ (circles) and SP_pH_ (triangles) at 10 mT.

The temperature dependence of the ZFC/FC magnetization curves of this series of SPION samples is presented in Figure 7 and Figure S11. At zero field (ZFC curve), the magnetic moments of the SPIONs are randomly distributed, showing a very low net magnetic moment.

**Figure 8:**
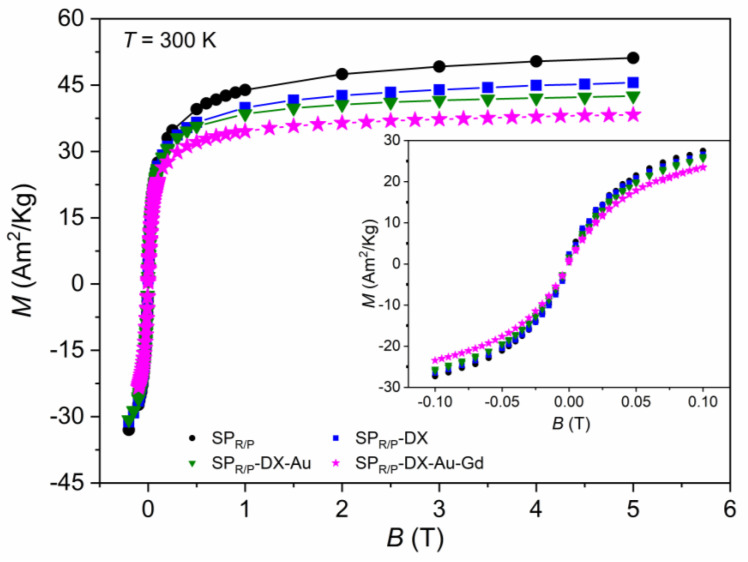
Magnetic field (B) dependence of magnetization (M) for M_R/P_ samples, SP_R/P_ (circles), SP_R/P_-Dx (squares), SP_R/P_-Dx-Au (triangles), and SP_R/P_-Dx-Au-Gd (stars), at 300 K.

On the other hand, when the samples are cooled in the presence of a magnetic field (FC curves), the magnetic moments of the particles align along the field direction, and we observe a stronger net moment. ZFC curves increase with temperature from 4 K up to 310 K as the magnetic moments gradually align throughout the measured temperature range. A superparamagnetic state, characteristic of particles with such small dimensions, is evidenced by the irreversibility of ZFC and FC curves and the observation of broad maxima of the ZFC curves, although with some particle size distribution. Larger nanoparticles require a higher temperature for the thermal energy to exceed the energy barrier and to behave as superparamagnets [37]. Estimated blocking temperatures T_B_ are shown in Table 3. SP_R/P_ exhibits a T_B_ of 85 K, indicating they have superparamagnetic behavior at RT. The fact that no coercivity (H_c_) and no magnetic remanence (M_R_) is observed at 300 K in the curves of the magnetic field dependence of the magnetization, M(B) up to 5 T (Figure 8), confirms superparamagnetic behavior at room temperature. At 10 K, a small coercive field, Hc, is present (Figure S12). The large surface-to-volume ratio commonly observed in SPIONs usually gives rise to high chemical activity and a tendency for the NPs to aggregate. In this way, the superparamagnetic behavior is a good advantage for their use in theranostic, suggesting no tendency to form clusters. 

As the SPIONs core gradually becomes more covered, the saturation of magnetization (Ms) values tends to decrease, from samples SP_R/P_ to SP_R/P_-Dx-Au-Gd (Figure 8) with values varying between 51.00 and 38.30 Am^2^/Kg, respectively, although the superparamagnetic behavior remains unchanged. The Ms value for the naked sample, SP_R/P_, is in good agreement with the literature on synthesized SPIONs through similar co-precipitation methods [14,20]. On the other hand, the saturation of magnetization for the completed covered sample, SP_R/P_-Dx-Au-Gd, decreased by 24.9% relative to its naked version; however, this Ms reduction is significantly lower compared with other reported values [36,37] where SPIONs after gold coating lost more than 30% of their Ms values. Therefore, the small Ms reduction that was obtained after the coating and functionalization processes may be a breakthrough in this research. Overall, these magnetic results ensure the use of such nanoparticles for biomedical applications.

At 10 K (Figure S12), quite below their T_B_, all samples exhibit higher saturation of magnetization and hysteresis loop as the field changes, meaning that at this temperature, it is necessary to apply a coercive field to reach a magnetization of zero, which reveals the ferrimagnetic behavior of the iron oxides nanoparticles.

#### 2.5.2. Samples Obtained with the Co-Precipitation with Controlled pH (SP_pH_)

Similar measurements were performed for samples prepared by the M_R/P_ method, and ZFC/FC cycles were performed for samples where SPIONs were obtained with controlled pH, as shown in Figure 7 and Figure S13. These measurements revealed blocking temperatures, T_B_, relatively higher than the previous, as expected considering the differences in their particle size according to TEM and XRD; samples obtained via M_R/P_ have smaller sizes than samples via M_pH_, and consequently, T_B_ occurs at lower temperatures. It is important to note the different paths of these ZFC/FC cycles, which are more pronounced in sample SP_pH_ compared with the correspondent sample SP_R/P_. An additional kink at a temperature lower than 100 K is observed, which is probably related to the Verwey transition characteristic of magnetite observed in the Mössbauer spectra. The fact that this occurs at temperatures lower than the Verwey transition temperature (observed at 122–125 K for bulk magnetite with the ideal stoichiometry) may be explained by several factors, among which are the partial oxidation and small size of the magnetite particles [14,20,57]. The narrow range of temperatures where this transition occurs in sample M_pH_, 33–38 K, is possibly related to the similar sizes of these nanoparticles (see Table 2). The absence of this kink in samples obtained from the M_R/P_ method can be due to the lower content of magnetite nanodomains as well as the smaller size distribution of the nanoparticles, as revealed by the structural and Mössbauer data.

**Figure 9:**
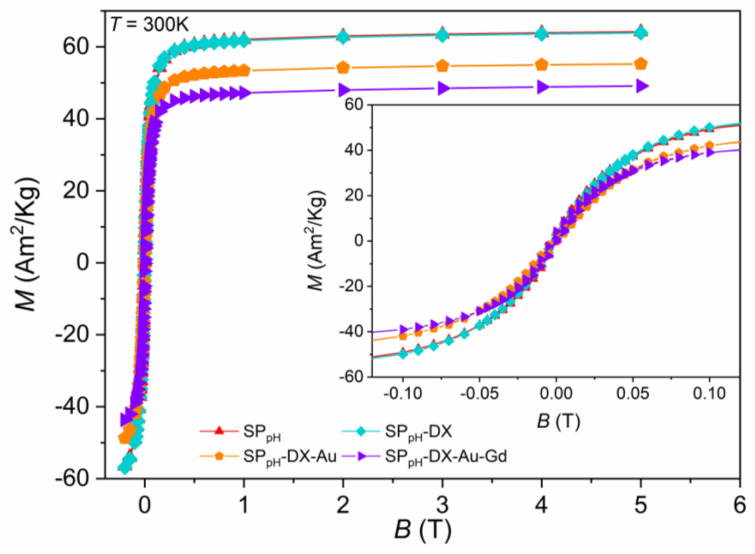
Magnetic field (B) dependence of magnetization (M) for M_pH_ samples, SP_pH_ (triangles), SP_pH_-Dx (diamonds), SP_pH_-Dx-Au (pentagons), and SP_pH_-Dx-Au-Gd (lying triangles), at 300 K.

Isothermal magnetization curves were obtained up to 5 T, at fixed temperatures, 300 K and 10 K (Figure 9 and Figure S14), to estimate the saturation of magnetization (Table 3) and to confirm superparamagnetism. At room temperature, a considerable value of Ms = 64.2 Am^2^/Kg for the naked version of SP_pH_ was obtained. This value is comparable with those reported in the literature for magnetite nanoparticles with similar average size and spherical shape [20,38,39]. As seen in Figure 8 and Figure 9, and by comparing the M_R/P_ samples with these new ones, type by type, one can notice that the SPIONs obtained by the M_pH_ method show higher Ms values confirming the above XRD and Mössbauer data lower oxidized spinel content, where a higher % of Fe_3_O_4_ was estimated. These good results validate this synthesis method as the one that may provide SPIONs with the best magnetic performance so far.

As expected, samples covered with Au and subsequently with Gd have their Ms values decreased by about 10% and 20%, respectively, although with a still significant magnetic signal of Ms, which is in accordance with other reports of synthesized SPIONs through co-precipitation methods [39,40,41,58]. The fact that no coercivity (H_c_) and no magnetic remanence (M_R_) are observed at 300 K on the isothermal curves shows, as referred to above, that they are superparamagnetic at room temperature. On the other hand, a small H_C_ appears at low temperature, namely 10 K (Figure S14), which reveals “blocking” of the SPIONs at low temperature, giving rise to the opening of a hysteresis loop.

### 2.6. Magnetic Hyperthermia

**Figure 10:**
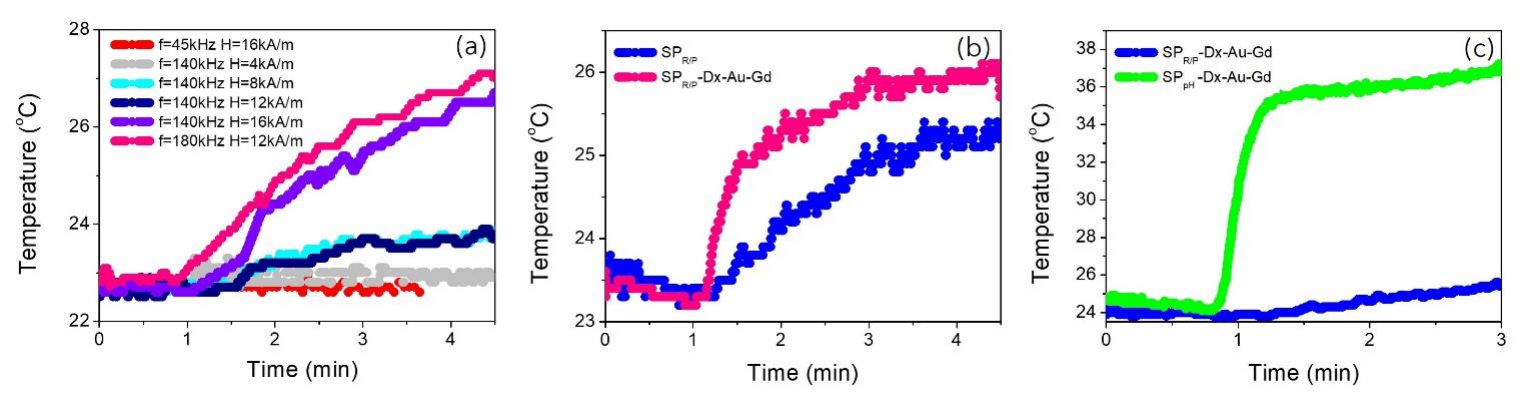
Heating performance of SPIONs samples: (**a**) sample SP_R/P_ measured at different conditions of frequency and field; (**b**) samples SP_R/P_ and SP_R/P_-DX-Au-Gd, measured at ƒ = 180 kHz and B = 12 kA/m (**c**) Samples SP_R/P_-DX-Au-Gd and SP_pH_-DX-Au-Gd, measured at ƒ = 140 kHz and B = 16 kA/m.

Naked SPIONs (sample SP_R/P_) were submitted to an alternated magnetic field with different amplitudes (4, 12, and 16 kA/m) and different frequencies (45, 90, 140, and 180 kHz). These acquired data include temperature stabilization (1 min) and the heating of the sample until temperature saturation, as shown in Figure 10a). The heating curves were fitted with the Box–Lucas method [59,60], and the obtained specific absorption rates (SARs) are summarized in Table 4. For the same frequency (140 kHz), the SAR value increases by increasing the intensity of the magnetic field. Increasing the intensity of the magnetic field means that more energy is being applied to the magnetic nanoparticles.

As a result, more heat is generated through magnetic relaxation processes. Also, a higher magnetic field intensity can lead to a greater alignment of magnetic moments in the nanoparticles with the field. This enhanced alignment also makes the nanoparticles more effective at absorbing and dissipating the energy from the alternating magnetic field (AMF) [61]. Maintaining a constant intensity for the magnetic field (12 kA/m) and varying the frequency (45, 90, and 140 kHz) applied to the sample SP_R/P_, the SAR value also significantly increases. This behavior can be associated with hysteresis losses or relaxation time [62]. At higher frequencies, the hysteresis cycles occur more rapidly, leading to increased hysteresis losses and, consequently, higher heat generation [63]. Also, if the frequency of the AMF matches or is close to the relaxation time of the nanoparticles, it can result in enhanced energy absorption and heat generation. 

When the SPIONs are functionalized with Dextran (SP_R/P_-DX), the SAR value decreases significantly, as shown in Table 4. Dextran coating increases the overall size of the SPIONs and affects their hydrodynamic behavior. Larger particles may experience reduced Brownian motion and less efficient heat generation in response to an alternating magnetic field, especially at low frequencies. This can contribute to a lower SAR.

When Gd is introduced in the SPIONs (sample SP_R/P_-DX-Au-Gd), the SAR values significantly increase (Figure 10b and Table 4). Gadolinium is a lanthanide element with strong paramagnetic properties. When Gd ions are introduced into SPIONs, they increase the overall magnetic moment of the nanoparticles. This enhanced magnetic moment leads to a more efficient response to an external magnetic field and contributes to higher heat generation, resulting in an increased SAR.

Both SPIONs doped with Gd and coated with Dextran and gold (SP_R/P_-Dx-Au-Gd and SP_pH_-Dx-Au-Gd) present the same behavior; however, the overall value of SAR is higher for SP_pH_-Dx-Au-Gd (Figure 10c). This sample presents the highest SAR value, up to a frequency of 140 kHz, and a high magnetic field (16 kA/m) due to the high dimensions of these nanoparticles, which are still in a superparamagnetic regime. The size of SPIONs plays a critical role in their response to an AMF. These larger nanoparticles present larger magnetic moments (as discussed in the previous sections) due to increased volume and magnetic properties. This resulted in higher energy absorption and, consequently, higher SAR.

### 2.7. Relaxitivity Studies

**Figure 11:**
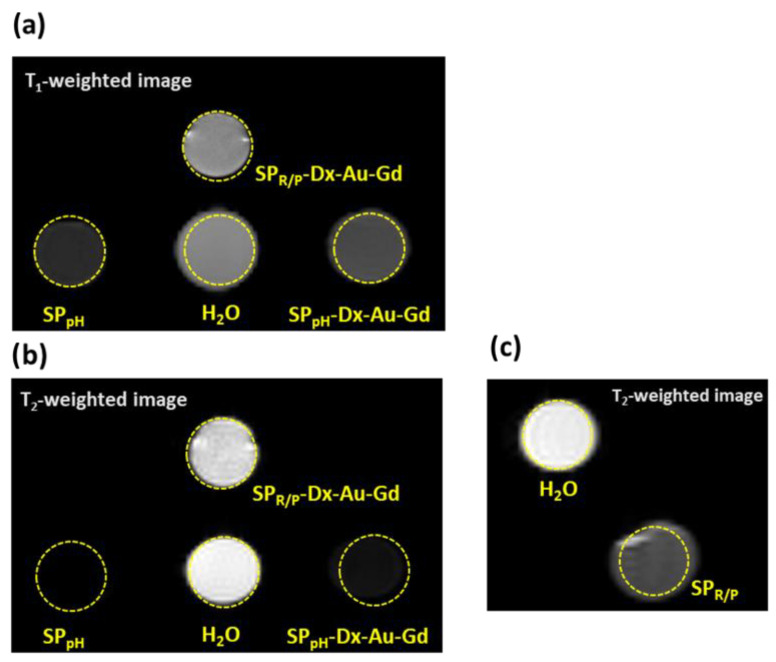
MR Images of phantom tubes containing samples SP_R/P_ and SP_R/P_-Dx-Au-Gd, and SP_pH_ and SP_pH_-Dx-Au-Gd, as well as water, acquired at 7 T. (**a**) T_1_–weighted images acquired at TE/TR = 21/626 ms. (**b**,**c**) T_2_–weighted images were acquired at TE/TR = 80/2000 ms and TE/TR = 8/3000 ms, respectively.

To assess the MRI potential application of the particles, we have imaged phantom tubes containing SPION samples in a 7 T Bruker scanner, Bruker, Wissembourg, France (300 MHz, room temperature, Figure 11) and measured the longitudinal (*r*_1_) and transverse (*r*_2_) relaxivities based on the T_1_ and T_2_ maps generated (Table 5, Tables S5 and S6). A sample of water was also measured to determine the diamagnetic contribution of the solvent, and this value was used to determine the relaxivity. Additionally, relaxivities were measured at 1.41 T (60 MHz, 25 °C), a magnetic field close to the clinically used ones. Samples 0.14 ± 0.04 mg/mL were prepared in water, and their Fe, Gd, and Au contents were measured by ICP-OES. The relaxivity values were calculated using the Gd concentration for the *r*_1_ determination and the Fe concentration for the *r*_2_. Due to the high impact of the presence of both metal ions within the same particle, the relaxivity values were also calculated considering the total concentration of metals (Fe + Gd). Tables S5 and S6 show all values. Table 5 also depicts the *r*_2_/*r*_1_ ratios calculated based on the separated and joint contributions of Fe and Gd.

The influence of several parameters in the relaxivity of SPIONs has been studied [64,65,66,67]. These include mainly size, shape and surface structure and modifications. As a first observation, the naked SPIONs have *r*_2_ relaxivities similar to other SPIONs reported in the literature and the same range as the commercially available Feridex or Resovist (120 and 186 mM^−1^s^−1^, respectively, 1.5 T) [64].

The presence of Au and Gd induced a decrease in the relaxivity of functionalized particles prepared by both methods, which is consistent with the M_s_ decrease observed between samples functionalized with Gd, SP_R/P_-Dx-Au-Gd and SP_pH_-Dx-Au-Gd, and the naked samples, SP_R/P_ and SP_pH_ (Table 3), and coating effects partially attributed to the presence of the diamagnetic layer of Dx, as reported in the literature [68,69].

It is also clear that the presence of Gd enables a longitudinal relaxivity contribution similar to or higher than the *r*_1_ relaxivities of the typical Gd-based MRI contrast agents in clinical use (3.4 to 4.6 mM^−1^s^−1^ at 1 T (42 MHz), 37 °C) [70,71]. SPIONs functionalized with Gd and/or gold NPs typically present *r*_1_ relaxivities that can vary between 4 and 40 mM^−1^s^−1^ [64,65,67,69,72,73]. Data reported in the literature are acquired under different conditions, namely different magnetic fields, which highly affect the relaxivity [71]. For example, at 7 T, Lip-DO3A@AuNP present a *r*_1_ of 4 mM^−1^s^−1^ [72] while Gd-Au-SPIONs have a *r*_1_ of 43.6 mM^−1^s^−1^ and a *r*_2_ of 123 mM^−1^s^−1^ [73]. 

Changes in the magnetic field are quite dramatic, and the impact on the *r_1_* and *r_2_* properties do not follow a similar pattern. While longitudinal relaxivities decrease with increasing field upon reaching a maximum at about 20–60 MHz (0.5–1.4 T), the transverse relaxivities also reach a maximum in the same region but then either reach a plateau or increase with an increase in the magnetic field, up until around 500 MHz (≈11 T) after which the values decrease again [74,75,76]. Our measurements are in accordance with the profiles observed before for SPIONs.

Typically, probes presenting a transverse-to-longitudinal relaxivity ratio (*r*_2_*/r*_1_) smaller than 2 are considered positive or T_1_-contrast agents. Ratios larger than 10 imply negative or T_2_- agents, while 2< *r*_2_*/r*_1_ <10 refers to potential dual-functional contrast agents [77,78]. Thus, sample SP_pH_-Dx-Au-Gd can be potentially used as a dual T_1_/T_2_ agent. Nevertheless, as the amount of iron is 10 to 20-fold higher than the gadolinium content, to better understand the impact in terms of contrast, the total concentration of metals ([Fe] + [Gd]) was used to re-calculate the *r*_1_ and *r*_2_ values as well as the corresponding *r*_2_/*r*_1_ ratio. The *r*_1_ values are highly affected, while the *r*_2_ ones do not change. Such a shift in the values has also been reported for the Gd-Au-SPIONs reported by Cheng et al. [73].

Figure 11 illustrates MRI T_1_- and T_2_-weighted images obtained from phantom tubes containing SPIONs studied, confirming the detectability of these samples in a 7 T MRI scanner. While the T2 effect seems more easily visualized, the T1-weighted images seem rather distorted, showing an unexpected visual contrast compared with the water tube.

Importantly, when comparing SPIONs prepared by the two methods, we observe that the co-precipitation one, M_pH_, leads to particles with higher relaxivities. This is, in fact, consistent with the higher percentage of iron(II) present in SP_pH_, which in turn leads to higher Ms values and better hyperthermia properties.

## 3. Materials and Methods

### 3.1. Materials

All chemicals and solvents were of reagent grade and were used without additional purification unless stated otherwise. The Milli-Q water was produced from a Millipore system Milli-Q ≥ 18 MΩcm (with a Millipak membrane filter of 0.22 μm). Iron(III) chloride hexahydrate (FeCl_3_·6H_2_O), iron(II) chloride tetrahydrate (FeCl_2_·4H_2_O), sodium sulfite (NaSO_3_), ammonia solution (NH_3_ aqua, 25%), hydrochloric acid (HCl, 37%), sodium hydroxide in pellets (NaOH), tetrachloroauric(III) acid trihydrate 99% (HAuCl_4_·3H_2_O), sodium borohydride (NaBH_4_), gadolinium(III) chloride (GdCl_3_·6H_2_O), and dextran (Dx) from Leuconostoc mesenteroides (average mol wt. 9.000–11.000) were commercially acquired from Aldrich Chemical ( St. Louis, MO, USA).

The ligand 2-[4,7-bis(carboxymethyl)-10-[2-(3-sulfanylpropanoylamino)ethyl]-1,4,7,10-tetrazacyclododec1-yl]acetic acid (TDOTA) was synthesized as previously reported.16

### 3.2. Synthesis of Naked SPIONs

#### 3.2.1. Reduction–Precipitation Process (M_R/P_)

The synthesis of naked SPIONs (SP_R/P_) was based on the method described by Matos et al. [14], consisting of Fe^3+^ reduction with Na_2_SO_3_ followed by Fe_3_O_4_ precipitation through titration with NH_3_ aqua. In a representative reaction, a solution of 4.5 g (16.67 mmol) of FeCl_3_.6H_2_O in 2.5 mL of HCl (2 M) and 2.5 mL of Milli-Q water was carefully added to a solution of 420 mg (3.33 mmol) of Na_2_SO_3_ in 3 mL of Milli-Q water and stirred at a moderate level at r.t. and P_atm_, for 30 min. A solution containing 125 mL of NH_3_ aqua (25% *w*/*w*) was then added dropwise to the previous solution. Due to the precipitation of Fe_3_O_4_, the color of the solution becomes dark during this phase. The final product was magnetically stirred for approximately 24 h, centrifuged at 4000 RPM for 10 min, and washed twice with Milli-Q water. After freeze–drying the reaction product, 644 mg of SPR/P were obtained.

#### 3.2.2. Co-Precipitation Process with Controlled pH (M_pH_)

SP_pH_ nanoparticles were obtained based on the strategy of Saraiva et al. [20], which consists of a standard co-precipitation method at room temperature, using as precursors ferrous and ferric chlorides in a molar ratio [Fe^3+^/Fe^2+^] = 1:2. In a typical reaction, two aqueous solutions of FeCl_3_.6H_2_O (0.01 M) and FeCl_2_.4H_2_O (0.02 M) were mixed. The resulting solution was de-aerated with nitrogen gas (N_2_) for 10 min to ensure the removal of oxygen during the co-precipitation process. To promote the precipitation of Fe_3_O_4_, NH_3_ aqua (25% *w*/*w*) was slowly added until the pH of the solution was close to 9.6. The instantaneously formed SPION suspension was filtered through a 0.22 μm membrane, washed with Milli-Q water, and the obtained precipitated SP_pH_ were freeze–dried.

### 3.3. Post-Synthesis of SPIONs Coating

#### 3.3.1. Dextran Coating

Briefly, in the typical reaction, a solution of 241 mg of Dx in 6 mL of NaOH (0.5 M) was slowly added to a naked SPIONs suspension (SP_R/P_ or SP_pH_) with a density of 8 mg/mL under intense magnetic stirring and maintained at 175 °C. After completing the addition, the solution was left under moderate magnetic stirring and at room temperature for 24 h. The resulting dextran-coated SPIONs (SP_R/P_-Dx and SP_pH_-Dx) were washed three times with new Milli-Q water to remove excess polymer and were lyophilized.

#### 3.3.2. Gold Coating

In a typical reaction, SP_R/P_-Dx or SP_pH_-Dx (209 mg) were then spread in Milli-Q water (30 mL) and added drop by drop to a solution of 96 mg HAuCl_4_·3H_2_O in 10 mL of Milli-Q water (0.028 M) at 70 °C. The color of the solution turns from dark brown to reddish brown after 15 min. Then, the pH of the solution was raised from 3 to around 9 by the addition of NaBH_4_ solution (0.37 M) and left to react for 5 h under moderate stirring. The final products (SP_R/P_-Dx-Au and SP_pH_-Dx-Au) were washed three times with Milli-Q water, centrifuged at 5000 rpm for 10 min, and lyophilized.

#### 3.3.3. Gadolinium Functionalization

In the final step, the SP_R/P_-Dx-Au and SP_pH_-Dx-Au were functionalized with gadolinium. For that, a solution of 78.6 mg of TDOTA16 was added to a suspension of 132 mg of SPIONs in 5 mL Milli-Q water and left to react for 7 h at room temperature. Then, 61 mg of GdCl_3_·6H_2_O in 3 mL of Milli-Q water was slowly added to the former solution and left to react for 18 h under moderate stirring. The pH of the solution drops from about 8 to near 6, confirming the complexation of Gd^3+^ to the nanoplatform. Finally, to isolate SP_R/P_-Dx-Au-Gd and SP_pH_-Dx-Au-Gd, the suspensions were placed in a dialysis membrane and dialyzed for two days in Milli-Q water, changing the water three times a day. After dialysis, the solutions were centrifuged (5000 rpm, 12 min), washed with Milli-Q water three times, and freeze–dried for 48 h.

### 3.4. Characterization of the SPIONs

#### 3.4.1. UV-Vis Spectrophotometry (UV-Vis)

The UV-Vis spectra of MILI-Q SPIONs dispersions with a concentration of 50 µg/mL were recorded at room temperature using a Varian Cary 50 UV/Vis spectrophotometer in a quartz cell (path length = 1 cm) in the range 200–800 nm.

#### 3.4.2. Attenuated Total Reflectance Fourier-Transform Infrared Spectroscopy (ATR-FTIR)

The Fourier transform infrared (FTIR) spectra were recorded at room temperature using an FTIR Nicolet iS50 spectrometer, an ATR diamond crystal accessory (Thermo Fisher Scientific, Madison, WI, USA), and KBr pellets. The spectra were obtained with a resolution of 4 cm^−1^ and 128 scans from 4000 to 400 cm^−1^.

#### 3.4.3. Powder X-ray Diffraction (PXRD)

The synthesized SPIONs were characterized by powder X-ray diffraction (PXRD) using a Bruker D2 Phaser diffractometer with a Cu Kα X-ray tube (monochromatic radiation, λ = 1.5406 Å). All the scans were obtained for 2θ between 10° to 80°, with an electrical high tension of 30 kV, a current of 10 mA, a step size of 0.02°, and a time per step of 10 s. The diffractograms were collected electronically and analyzed using the DIFFRAC.EVA pattern processing software (2006–2022 MiniTool Power Data Recovery Free Edition). The estimates of the size of the SPIONs crystallites were obtained by Scherrer’s equation:

(1) $$D_{XRD}=\frac{K\lambda }{\beta Cos(\theta )}$$

where *D* stands for the value of the crystallite size (nm), *λ* the X-ray wavelength (0.154 nm in the present case), *K* the Scherrer constant, *θ* the Braggs angle of the most intense diffraction peak, and *β* the full width at the half maximum of this peak in radians [21]. *K* values are within the range of 0.62–2.08, and when no information on the crystal shape is available, the value for spherical particles, *K* = 0.9, is generally used [25]. The resulting crystallographic patterns were analyzed with the help of the software DIFFRAC.EVA, where the crystallite size and chemical matching were determined, combined with some corrections to the curve, such as background subtracting and smoothing of the lines. The intensity of each peak in the patterns was used as an estimate of the relative percentage of each compound, which was then compared with other quantifying techniques. The final graphs were then plotted with the help of the software Origin (version 2019).

#### 3.4.4. Transmission Electron Microscopy (TEM)

The size and the shape of the selected multifunctional nanoplatforms were characterized by TEM at Instituto Gulbenkian de Ciência (IGC). Samples were prepared by dipping a 300-mesh carbon-coated copper grid into a solution containing the SPIONs sample previously sonicated, followed by air drying at room temperature for 5 min. Images were obtained on a FEI Tecnai G2 Spirit BioTWIN transmission electron microscope. Software ImageJ (version 1.35J) was used to process the obtained images to determine the average size and shape of the SPIONs. Size dispersion was calculated using Equation (2):

(2) $$\sigma \;(\%)=\frac{Standard\;Deviation}{Average\;NPs\;size}\times 100$$

#### 3.4.5. Dynamic Light Scattering (DLS) and Zeta-Potential

DLS measurements were conducted utilizing a Malvern Zetasizer Nano ZS (Malvern Instruments Ltd., Worcestershire, UK) with a 633 nm He-Ne laser, operating at a 173° angle. Data collection and analysis were performed using the Dispersion Technology Software (DTS) version 5.10 from Malvern. Each sample (600 µL) was measured in low-volume semi-micro disposable sizing cuvettes (Fisher Scientific, USA) with a 10 mm path length. Triplicate measurements were taken at a distance of 4.65 mm from the cuvette wall with an automatic attenuator. Fifteen runs of 10 s each were conducted for every sample. The size distribution, Z-average diameter, and polydispersity index (PDI) were derived from the autocorrelation function using the “general purpose mode” for all nanoparticle samples. The default parameters, including a filter factor of 50%, a lower threshold of 0.05, and an upper threshold of 0.01, were employed. Zeta potential measurements were conducted in duplicate using water as a dispersant and the Huckel model. Each sample underwent 20 runs in auto-analysis mode.

#### 3.4.6. Determination of the Iron, Gold, and Gadolinium Content

The iron (Fe), gold (Au), and gadolinium (Gd) contents of the obtained nanoparticles were quantified using Inductively Coupled Plasma Optical Emission Spectrometry (ICP-OES). Spectrometric (ICP-OES) measurements were performed using an Agilent Technologies 5800 VDV ICP-OES. Fe, Gd, and Au standard solutions were prepared in about 5–10% Aqua regia matrix (HNO_3_:HCl, 1:3). A 200 µL sample of each NP solution prepared were digested following the protocol: (1) evaporation to dryness, (2) re-dissolved in 1 mL of concentrated Aqua Regia and evaporated to dryness, (3) addition of 100 µL of 30% H_2_O_2_ + 100 µL of Milli-Q water are then added and evaporated to dryness and finally retaken in 5 mL of about 5–10% Aqua Regia for ICP measurements. The quantitative determinations were performed using the most accurate wavelength: 342.246 nm for Gd, 259.940 nm for Fe, and 267.594 nm for Au. All the measurements were performed in triplicate.

#### 3.4.7. Mössbauer Spectroscopy

Transmission Mössbauer spectra of the NP samples were taken at room temperature and 4 K using a velocity versus time triangular wave form, a Wissel (Starnberg, Germany) spectrometer, and a 25-mCi ^57^Co source in Rh matrix. Isomer shift values, IS, are given relative to the α-Fe foil used to calibrate the velocity scale. The 4 K measurements were obtained with the sample dipped in liquid He in a Janis (Westerville, OH, USA) bath cryostat, model SVT-400. These data were analyzed by fitting Lorentzian lines using a non-linear least-squares computer method [20]. During the spectra analysis, the relative areas and line widths of both peaks in a quadrupole doublet and of peak pairs 1–6, 2–5, and 3–4 in a magnetic sextet were kept equal. The histogram method was used to fit distributions of magnetic splittings [79].

#### 3.4.8. SQUID Magnetometry

Static DC magnetic measurements were obtained using a 6.5 T SQUID (Superconducting Quantum Interference Device) magnetometer from Cryogenic Ltd. London, UK. To assess the saturation of magnetization (M_s_) and the superparamagnetic behavior at room temperature, isothermal curves were taken at temperatures of 300 K and 10 K for magnetic fields up to 5 T. The mean magnetic diameter (D_mag_) of the nanoparticles was estimated considering the *M*(*B*) curves obtained at 300 K through Equation (3):

(3) $$D_{mag}={\left(\frac{18K_{b}T{\left(\frac{dM}{dB}\right)}_{\left(B\;\to 0\right)}}{\pi \rho {M_{s}}^{2}}\right)}^{\frac{1}{3}}$$

where *K_b_* is the Boltzmann constant, which is equal to 1.381 × 10^−23^ m^2^Kg/s^2^K, *T* is the temperature in Kelvin, ${\left(\frac{dM}{dB}\right)}_{\left(B\;\to 0\right)}$ is the derivative of the first order of the curve *M*(*B*) at the point B = 0, M_s_ is the saturation magnetization, and ρ is the mass density of the iron oxide structure.

Temperature dependence of the magnetization within the range of 5–320 K in the presence of an external magnetic field of 100 Oe was also measured. These data were collected at increasing temperatures, from 10 to 300 K, after zero-field cooling and field cooling (ZFC/FC) cycles.

#### 3.4.9. Magnetic Hyperthermia

The heating efficiency of SPION dispersion in water ([Fe] = 4 mM/mL) was assessed using an alternating magnetic field (AMF) generated by AC GEN from Nanotech Solutions. The AMF had a frequency from 45 to 180 kHz and a magnetic field strength from 5 to 200 Oe, applied for a duration of 5 min (maximum H.f = 2.9 × 10^9^ Am^−1^s^−1^). These experimental results conform to the safety guidelines proposed by Hergt and Dutz, adhering to the criterion of H.f = 5 × 10^9^ Am^−1^s^−1^ for potential clinical applications of AMF. A sample volume of 500 μL was introduced into Eppendorf tubes and individually positioned within the magnetic coils. Temperature changes were continuously monitored and recorded every second using a thermal infrared camera located on the top of the coil cavity. The specific absorption rate (SAR) was calculated using the Box–Lucas method [59,60].

#### 3.4.10. MRI Phantoms and Relaxometric Measurements

MR images of tubes containing water and the SPIONs solutions (0.14 ± 0.04 mg/mL) were acquired on a 7 T Pharmascan 70/16 superconducting magnet (Bruker, Wissembourg, France) equipped with a shielded gradient set (230 mT m^−1^ maximum gradient amplitude) and a transmit-receive quadrature 38 mm inner diameter coil (Bruker BioSpin, Wissembourg, France). Two RARE sequences (rapid spin echo, factor 8) were used based on the following parameters: TE = 10, 20, 30, 40, and 50 ms and TR = 5.5, 3.0, 1.5, 0.8, 0.4, and 0.2 s. or TE = 8, 25, 41, 58, and 75 ms for the samples with very low T2. Images were registered based on 1.0 mm thick slices at 156 × 156 µm^2^ resolution, 128 × 128 matrix using a field of view of 4.0 × 4.0 cm. T1 and T2 values were fitted based on maps created with Paravision 5.0. All measurements were performed at room temperature, and a tube containing water was measured under the same conditions for visual comparison and as a diamagnetic contribution at 7 T. 

Longitudinal and transverse proton relaxivities were also measured at 60 MHz (1.41 T) and 25 °C on a Bruker Minispec relaxometer. 

The Fe and Gd concentrations were assessed by ICP-OES (see above).

## 4. Conclusions

We have thoroughly investigated and comprehensively characterized a series of nanoplatforms based on superparamagnetic iron oxide nanoparticles (SPIONs) prepared by two distinct methods. The magnetic efficacy, provided by key parameters such as magnetization, magnetic hyperthermia, and magnetic relaxivity, gave different results when comparing these two processes. Taken together, our findings indicate that both reduction–precipitation (M_R/P_) and co-precipitation at controlled pH (M_pH_) methods lead to particles with a convenient average size, 9 nm, well suitable for biomedical applications. However, the M_pH_ method produces nanoparticles with improved magnetic properties, consistent with a higher Fe content present in magnetite domains as deduced from Mössbauer spectroscopy, rendering them better suited for theranostic applications. In addition to maintaining magnetic properties, SPIONs coated with gold (Au) and gadolinium (Gd) exhibited excellent Specific Absorption Rate (SAR) and Magnetic Resonance Imaging (MRI) properties. Those obtained by the M_pH_ method had a better performance, namely significantly higher SAR values and improved relaxivities. This suggests their potential as dual T_1_/T_2_ contrast agents, thereby demonstrating high efficacy for magnetic hyperthermia applications in these nanoplatforms.

## Figures and Tables

**Table 1:** Samples, acronym, and their respective synthetic plan.

Sample	Description	Precursor
SP_R/P_	SPIONs obtained by reduction–precipitation	FeCl_3_·6H_2_O/Na_2_SO_3_
SP_R/P_-Dx	SP_R/P_ coated with dextran	SP_R/P_
SP_R/P_-Dx-Au	SP_R/P_-Dx coated with gold	SP_R/P_-Dx
SP_R/P_-Dx-Au-Gd	SP_R/P_-Dx-Au functionalized with Gd^3+^	SP_R/P_-Dx-Au
SP_pH_	SPIONs obtained by co-precipitation with controlled pH	FeCl_3_·6H_2_O/FeCl_2_·4H_2_O
SP_pH_-Dx	SP_pH_ coated with dextran	SP_pH_
SP_pH_-Dx-Au	SP_pH_-Dx coated with gold	SP_pH_-Dx
SP_pH_-Dx-Au-Gd	SP_pH_-Dx-Au functionalized with Gd^3+^	SP_pH_-Dx-Au

**Table 2:** Measurements of sizes obtained by PXRD, TEM, and DLS, and zeta potential, ζ, of all samples. Crystallite size, D_XRD,_ particle size, D_TEM_, and associated size dispersion, σ, magnetic nanoparticles diameter, Dmag, and hydrodynamic mean size, D_H_, for each sample.

Sample	Crystallite Size—D_XRD_ (nm)	Particle Size—D_TEM_ (nm)	Size Dispersion—σ (%)	Particle Size—Dmag (nm)	Hydrodynamic Size—D_H_ (nm) (PDI)	Zeta Potential (mV)
SP_R/P_	9.0 ± 0.6	10.0 ± 2.1	20.94	10.51	52.5 (0.6)	40.3 ± 8.3
SP_R/P_-Dx	7.4 ± 0.9	9.6 ± 1.5	27.58	11.00	352.4 (0.4)	−6.2 ± 7.6
SP_R/P_-Dx-Au	9.8 ± 0.8	10.9 ± 2.7	24.84	11.68	47.4 (0.6)	−19.1 ± 6.0
SP_R/P_-Dx-Au-Gd	9.4 ± 0.7	11.0 ± 2.8	24.22	12.28	127.9 (0.3)	35.4 ± 8.5
SP_pH_	10.0 ± 0.5	12.2 ± 2.5	20.84	10.09	25.4 (0.5)	43.8 ± 10.0
SP_pH_-Dx	12.0 ± 0.6	16.5 ± 4.1	24.80	9.46	136.1 (0.6)	−15.1 ± 1.3
SP_pH_-Dx-Au	18.2 ± 0.9	19.4 ± 5.6	28.62	9.65	28.2 (0.7)	−21.5 ± 0.7
SP_pH_-Dx-Au-Gd	18.3 ± 1.3	20.0 ± 5.0	25.18	10.85	42.5 (0.5)	−16.7 ± 0.7

**Table 3:** Magnetic parameters for all the studied samples.

	T_B_	T = 300 K	T = 10 K		
Samples	K	$M_{s}\;(Am^{2}/Kg)$	$M_{s}\;(Am^{2}/Kg)$	$M_{r}\;(Am^{2}/Kg)$	$H_{C}\;(T)$
SP_R/P_	85	51.00	60.59	10.12	0.018
SP_R/P_-Dx	72	45.50	53.60	10.90	0.018
SP_R/P_-Dx-Au	87	42.54	48.32	8.83	0.017
**SP_R/P_-Dx-Au-Gd**	90	38.30	45.20	7.76	0.016
SP_pH_	91	64.20	74.90	16.00	0.013
SP_pH_-Dx	82	63.90	73.68	14.00	0.014
SP_pH_-Dx-Au	132	55.26	64.47	12.50	0.018
SP_pH_-Dx-Au-Gd	140	49.16	64.02	14.00	0.012

**Table 4:** Specific absorption rate, SAR, values of samples at different frequencies, and applied magnetic fields **H**.

	**H (kA/m)**	**4**	**12**	**16**	**16**	**16**	**12**
**Sample**	**F (kHz)**	140	140	140	90	45	180
SP_R/P_		424	794	934	872	513	997
SP_R/P_-Dx		-	0	0	-	-	247
**SP_R/P_-Dx-Au-Gd**		-	375	4726	-	-	3588
SP_pH_		0	607	730	-	2023	1860
SP_pH_-Dx-Au		-	2970	57,082	-	-	32,143
SP_pH_-Dx-Au-Gd		-	8970	97,673	-	-	12,991

**Table 5:** Longitudinal (*r*_1_) and transverse (*r*_2_) relaxivity values determined for samples of the SPIONs at 300 MHz (room temperature, RT) and *r*_2_/*r*_1_ ratio.

Sample	7 T (300 MHz, RT)/mM^−1^s^−1^				1.41 T (60 MHz, 25 °C)/mM^−1^s^−1^			
	*r*_1_	*r*_2_	*r*_2_/*r*_1_	*r*_2_/*r*_1_ (Gd + Fe)	*r*_1_	*r*_2_	*r*_2_/*r*_1_	*r*_2_/*r*_1_ (Gd + Fe)
SP_R/P_	---	122	---	340	---	61.8	---	35
**SP_R/P_-Dx-Au-Gd**	5	10	2	23	5.5	7.7	1.4	29
SP_pH_	---	186	---	240	---	169.7	---	22
SP_pH_-Dx-Au-Gd	55	56	1	282	23.2	11.7	0.5	123

## Data Availability

The data are contained within the article and Supplementary Material.

## Supplementary Materials

The following supporting information can be downloaded at: https://www.mdpi.com/article/10.3390/molecules29081824/s1, Figure S1: UV-Vis spectra of MR/P samples; Figure S2: ATR-FTIR spectra of Dextran (pink) and MR/P samples, SPR/P (blue), SPR/P-Dx (yellow), SPR/P-Dx-Au (orange) SPR/P-Dx-Au-Gd (red). Figure S3 and Figure S4: Histograms of the size distribution by number of all samples by DLS analysis. Figure S5: Powder diffractogram of coated samples SPR/P-Dx (left) and SPR/P-Dx-Au (right). Figure S6: Powder diffractogram of coated samples SPpH-Dx (left) and SPpH-Dx-Au (right). Figure S7: Transmission electron microscopy images of the SPIONs respective size histogram: Top: SPR/P-Dx; bottom: SPR/P-Dx-Au. Figure S8: Transmission electron microscopy images of the SPIONs respective size histogram: Top: SPpH-Dx; bottom: SPpH-Dx-Au. Figure S9: Mössbauer spectra of SPR/P (left) and SPR/P-Dx-Au (right) taken at different temperatures. The lines over the experimental points are the calculated curves. The estimated parameters are collected in Table S3. Figure S10: Room temperature Mössbauer spectra of (a) SPpH-Dx (b) SPpH-Dx-Au and (c) SPpH-Dx-Au-Gd samples. Calculated lines on the experimental points are the sum of three sextets (see Table S3). Figure S11: Temperature dependence of the zero-field cooling (ZFC) and field cooling (FC) magnetization for samples, SPR/P-Dx, at 10mT (squares), SPR/P-Dx-Au, at 50 mT (down triangles) and SPR/P-Dx-Au-Gd, at 50 mT (stars). Figure S12: Magnetic field (*B*) dependence of magnetization (*M*) at 10 K for SPR/P-based samples, SPR/P (circles), SPR/P-Dx (squares), SPR/P-Dx-Au (down triangles) and SPR/P-Dx-Au-Gd (stars). Figure S13: Temperature dependence of the zero-field cooling (ZFC) and field cooling (FC) magnetization (at 10 mT) for SPpH-based samples, SPpH-DX (diamonds), SPpH-DX-Au (pentagons), and SPpH-DX-Au-Gd (lying triangles). Figure S14: Magnetic field (*B*) dependence of magnetization (*M*) at 10 K for SPpH-based samples, SPpH (triangles) SPpH-DX (diamonds), SPpH-DX-Au (pentagons), and SPpH-DX-Au-Gd (lying triangles). Table S1: Absorption peaks observed in the UV-Vis Spectra of the SPIONs. Table S2: Most Significant absorption bands observed in the FTIR Spectra of the SPIONs and Dextran. Table S3: Hydrodynamic size values for all samples. Table S4: Estimated parameters from the Mössbauer spectra of selected SPIONs samples at room temperature and at 4 K. Tables S5 Longitudinal (*r*1) and transverse (*r*2) relaxivity values determined for selected samples at 300 MHz, 7 T, room temperature, Fe and Gd concentrations, and *r*2/*r*1 ratios. Table S6. Longitudinal (*r*1) and transverse (*r*2) relaxivity values determined for selected samples at 300 MHz, 1.41 T, room temperature, Fe and Gd concentrations, and *r*2/*r*1 ratios.
