# Supplementary material for: Influence of SPION Surface Coating on Magnetic Properties and Theranostic Profile
Source: Molecules. 2024 Apr 17;29(8):1824. doi: 10.3390/molecules29081824 (PMC11052394; doi:10.3390/molecules29081824)
Supplement: Supplementary file 1 [file molecules-29-01824-s001.zip › molecules-2957691-supplementary.pdf]

Supplementary Materials

Influence of SPIONs Surface Coating on Magnetic Properties and Theranostic Profile

Vital Cruvinel Ferreira-Filho [1], Beatriz Morais [1], Bruno J. C. Vieira [1], João Carlos Waerenborgh [1], Maria João Carmezim [2,3], Csilla Noémi Tóth [4], Sandra Même [4], Sara Lacerda [4], Daniel Jaque [5], Célia T. Sousa [6], Maria Paula Cabral Campello [1,*] and Laura C. J. Pereira [1,*]

[1] Centro de Ciências e Tecnologias Nucleares, DECN, Instituto Superior Técnico, Universidade de Lisboa, E.N. 10, km 139,7, 2695-066 Bobadela LRS, Portugal; vital.filho@ctn.tecnico.ulisboa.pt (VFF); beatriz.morais@ctn.tecnico.ulisboa (BM); brunovieira@ctn.tecnico.ulisboa.pt (B.J.C.V.); jcarlos@ctn.tecnico.ulisboa.pt (J.C.W.); pcampelo@ctn.tecnico.ulisboa.pt (MPCC); lpereira@ctn.tecnico.ulisboa.pt (L.C.J.P.).

[2] Centro de Química Estrutural-CQE, DEQ, Instituto Superior Técnico, Universidade de Lisboa, Av. Rovisco Pais, 1049-001 Lisboa, Portugal; maria.carmezim@estsetubal.ips.pt (MJC).

[3] ESTSetúbal, CDP2T, Instituto Politécnico de Setúbal, Setúbal, Portugal; maria.carmezim@estsetubal.ips.pt (MJC).

[4] Centre de Biophysique Moléculaire, CNRS, UPR 4301, Université d'Orléans, Rue Charles Sadron,45071 Orléans CEDEX 2, France; csilla-noemi.garda-toth@cnrs-orleans.fr (CGT); sandra.meme@cnrs-orleans.fr (SM); sara.lacerda@cnrs-orleans.fr (SL).

[5] Departamento de Física de Materiales, Universidad Autonoma de Madrid, Avda. Francisco Tomás y Valiente 7, 28049 Madrid, Spain; daniel.jaque@uam.es (DJ).

[6] Departamento de Física Aplicada, Universidad Autonoma de Madrid, Avda. Francisco Tomás y Valiente 7, 28049 Madrid, Spain; celia.tsousa@uam.es (CTS).

* Correspondence: pcampelo@ctn.tecnico.ulisboa.pt (MPCC); lpereira@ctn.tecnico.ulisboa.pt (L.C.J.P.).; Tel.: +351219946233 (MPCC); Tel.:+ 351219946259 (LCJP).

Contents **Page**

1. UV-Vis spectrophotometry……………………………………………………..…….S2
2. Attenuated Total Reflectance Fourier-Transform Infrared Spectroscopy (ATR-FTIR)…...…………………….………………………………………………………..….S3
3. Dynamic Light Scattering (DLS) and Zeta-Potential……………………………………S4
4. Powder X-ray diffraction (PXRD) ………………………...S7
5. Transmission Electron Microscopy (TEM)………………………………………………S8
6. Mössbauer Spectroscopy…………………………………………………………..….S9
7. Magnetic Measurements…………..……………………………………………..……S12
8. Relaxitivity Studies………………………………………………………………………S14

1. *UV-Vis Spectrophotometry*

Table S1. Absorption peaks observed in the UV-Vis Spectra of the SPIONs

Sample	Wavelength (nm)	
$SP_{R/P}$	244	326
$SP_{R/P}$-Dx	248	388
$SP_{R/P}$-Dx-Au	390	543
$SP_{R/P}$-Dx-Au-Gd	394	544
SP_{pH}	251	320
SP_{pH}-Dx	229	381
SP_{pH}-Dx-Au	385	530
SP_{pH}-Dx-Au-Gd	395	540
Dextran	275	

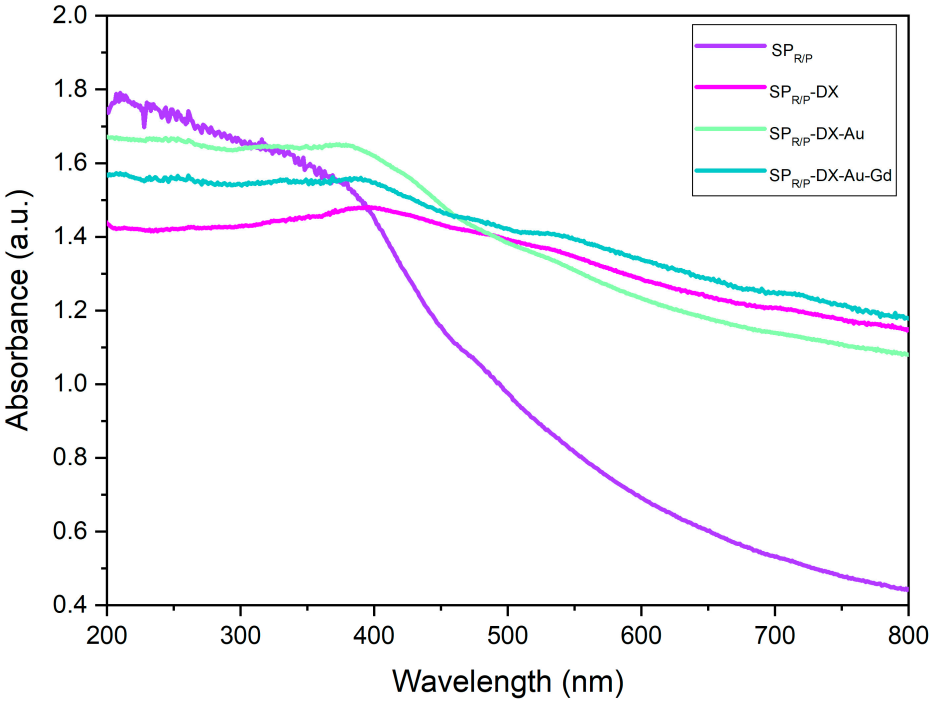

Figure S1. UV-Vis spectra of $M_{R/P}$ samples.

2. *Attenuated Total Reflectance Fourier-Transform Infrared Spectroscopy (ATR-FTIR)*

Table S2. Most Significant absorption bands observed in the FTIR Spectra of the SPIONs and Dextran.

**Sample**	**Wavenumber (cm^{-1})**								
	H_2O	Dextran		H_2O	Dextran		SPIONs		
SP$_{R/P}$	3417			1636			879	629	587
SP$_{R/P}$-Dx	3374	2922	2849	1624	1152	1018	890	620	576
SP$_{R/P}$-Dx-Au	3418	2918	2849	1634	1106	1017	890	627	573
SP$_{R/P}$-Dx-Au-Gd	3420	2920	2847	1635	1098	1025		625	590
SP$_{pH}$	3423			1634			863		590
SP$_{pH}$-Dx	3422	2923	2853	1637	1110	1016	866	617	579
SP$_{pH}$-Dx-Au	3422	2922	2849	1637	1111	1013	871	617	581
SP$_{pH}$-Dx-Au-Gd	3419	2928	2884	1634	1111	1051	840	618	590
Dextran	3404	2920	2881	1643	1157	1013			

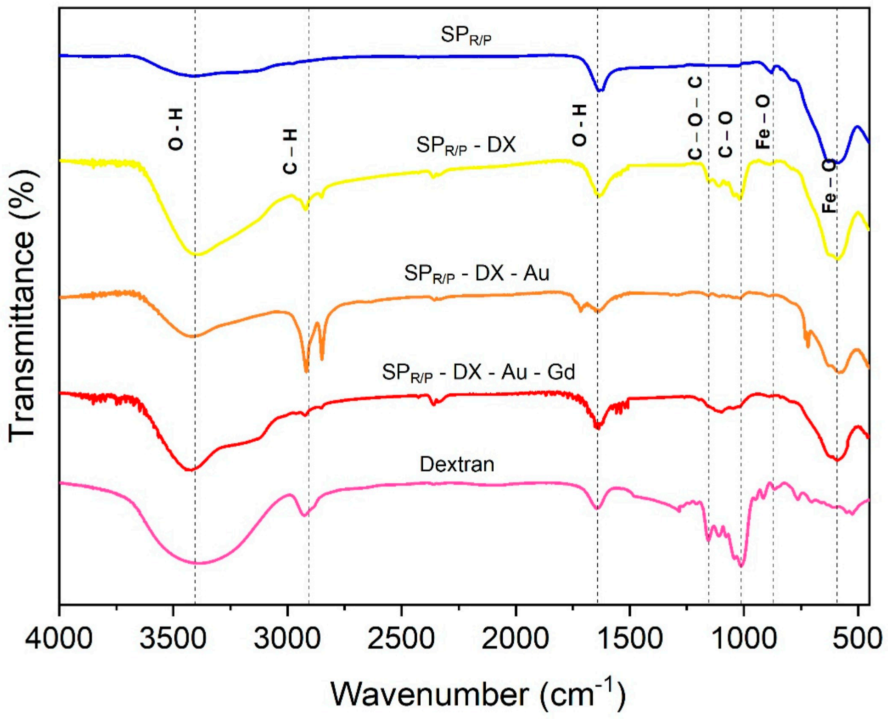

Figure S2. ATR-FTIR spectra of Dextran (pink) and M$_{R/P}$ samples, SP$_{R/P}$ (blue), SP$_{R/P}$-Dx (yellow), SP$_{R/P}$-Dx-Au (orange) SP$_{R/P}$-Dx-Au-Gd (red).

3. *Dynamic Light Scattering (DLS) and Zeta-Potential*

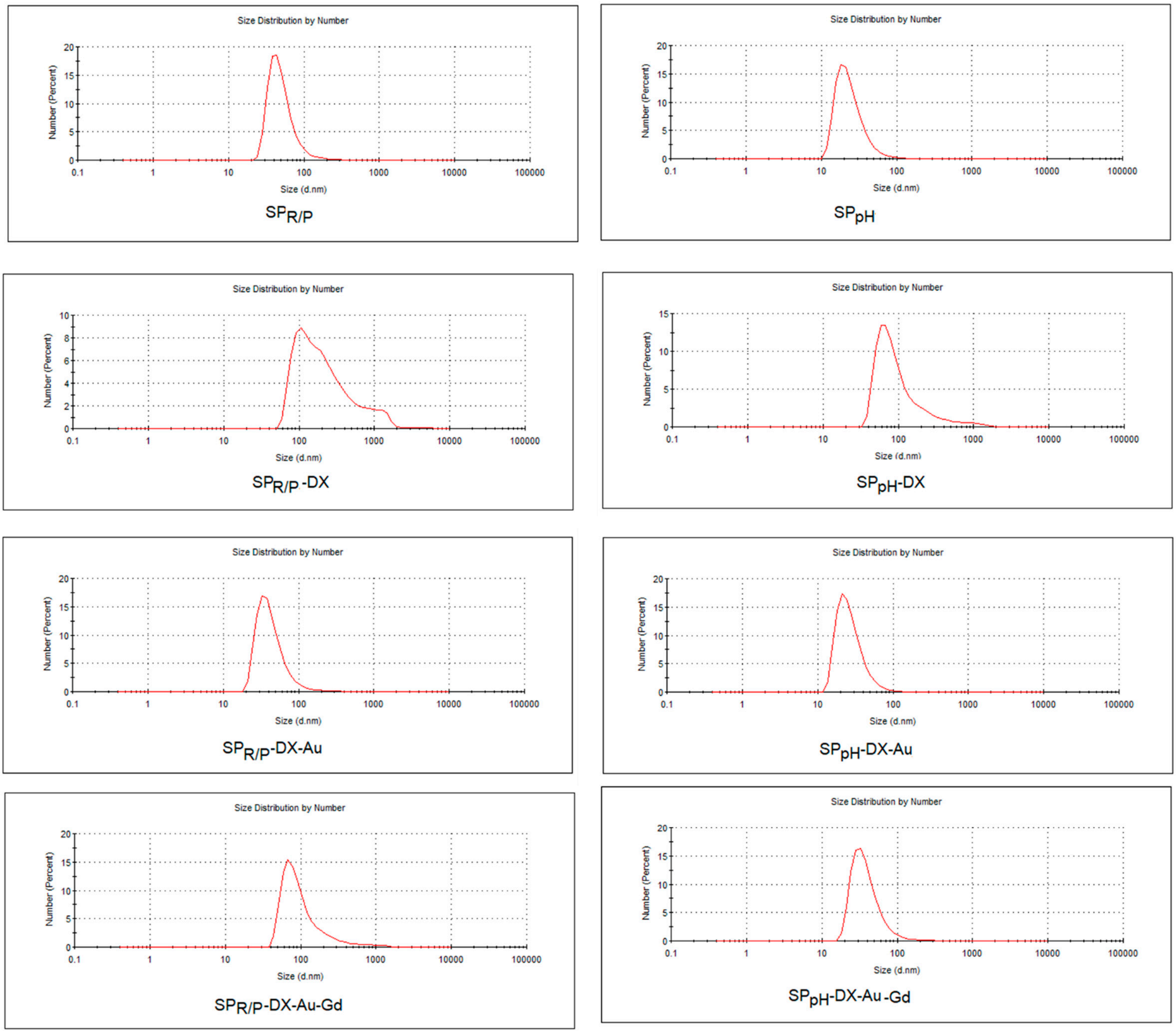

Figure S3. Histograms of the size distribution by number of all samples by DLS analysis.

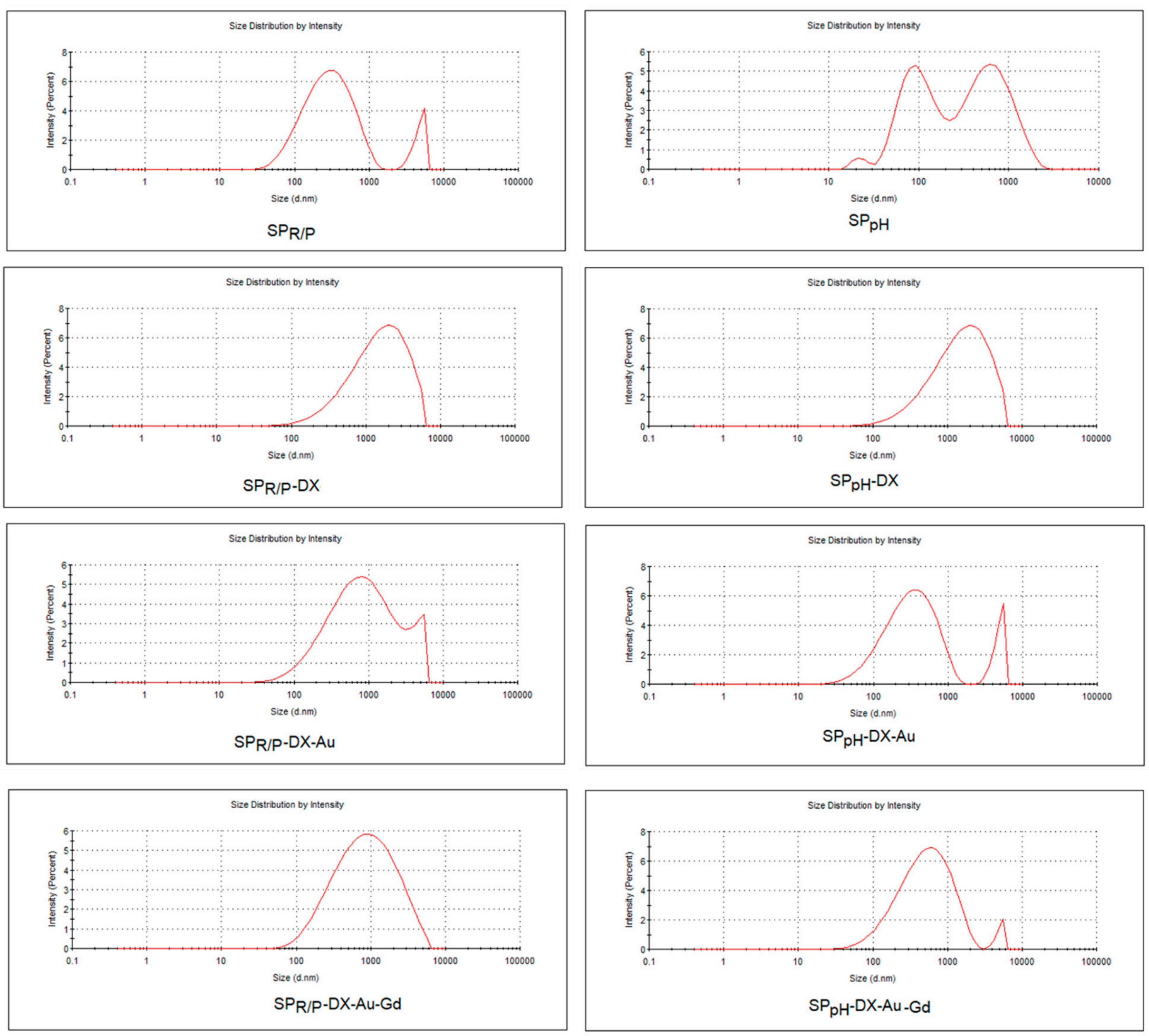

Figure S4. Histograms of the size distribution by intensity of all samples by DLS analysis.

Table S3. Hydrodynamic size values for all samples.

		Hydrodynamic size		
		INTENSITY		
**Sample**	**Z Average (d.nm)**	**Peak 1 (%)**	**Peak 2 (%)**	**Peak 3 (%)**
$SP_{R/P}$	289.4	343.3 (88.6)	4705 (11.4)	
	317.6	308.6 (82.8)	4640 (17.2)	
$SP_{R/P}$-Dx	1087	905.9 (64.9)	4215 (35.1)	
	1364	896.9 (53.0)	3974 (47.0)	
$SP_{R/P}$-Dx-Au	628.6	947.9 (85.3)	4321 (14.7)	
	581.3	1193 (100)		
$SP_{R/P}$-Dx-Au-Gd	575.8	1191 (100)		
	570.6	972.5 (100)		
SP_{pH}	166.6	700 (54.6)	107.2 (43.2)	23.27 (2.2)
	159.4	414.2 (90.4)	37.87 (9.6)	
SP_{pH}-Dx	999.3	947.8 (65.6)	3985 (34.4)	
	1024	1024 (65.5)	3761 (34.5)	
SP_{pH}-Dx-Au	333.0	379.9 (86.2)	4795 (13.8)	
	316.3	334.3 (86.8)	4885 (13.2)	
SP_{pH}-Dx-Au-Gd	426.9	626.4 (96.0)	5047 (4.0)	
	422.1	568.1 (94.1)	4971 (5.9)	

4. *Powder X-ray diffraction (PXRD)*

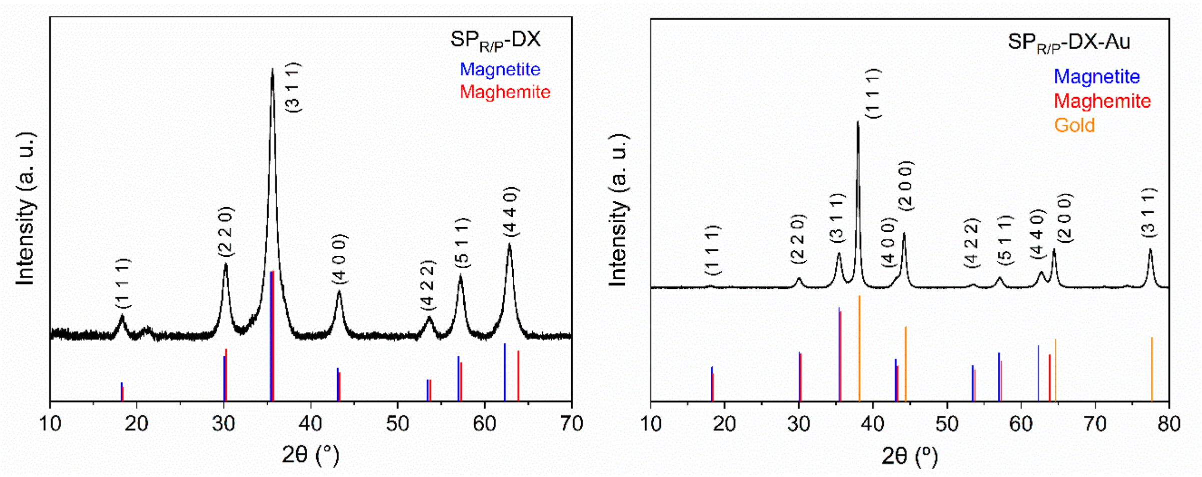

Figure S5. Powder diffractogram of coated samples $SP_{R/P}$-Dx (left) and $SP_{R/P}$-Dx-Au (right).

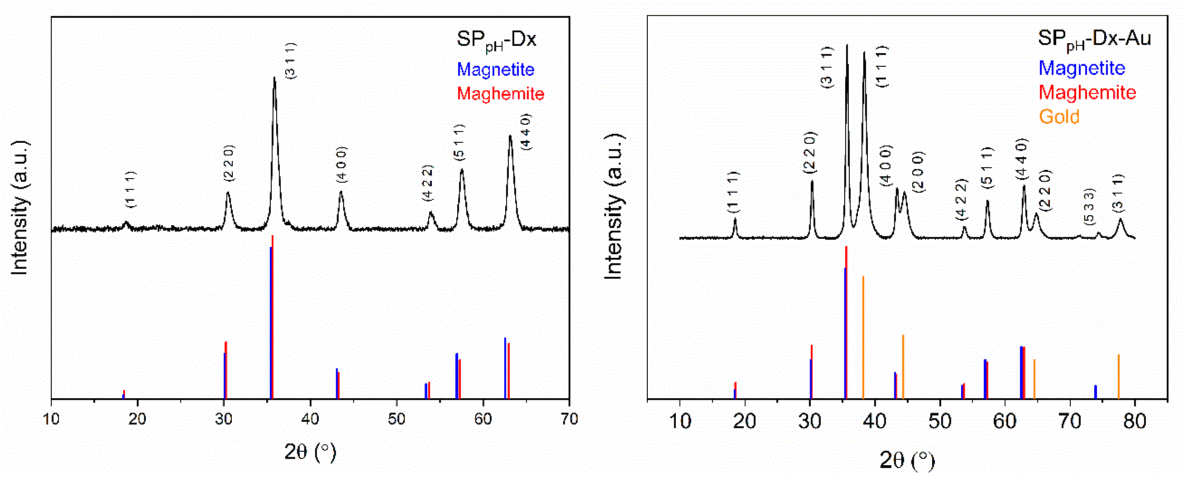

Figure S6. Powder diffractogram of coated samples SP_{pH}-Dx (left) and SP_{pH}-Dx-Au (right).

5. *Transmission Electron Microscopy (TEM)*

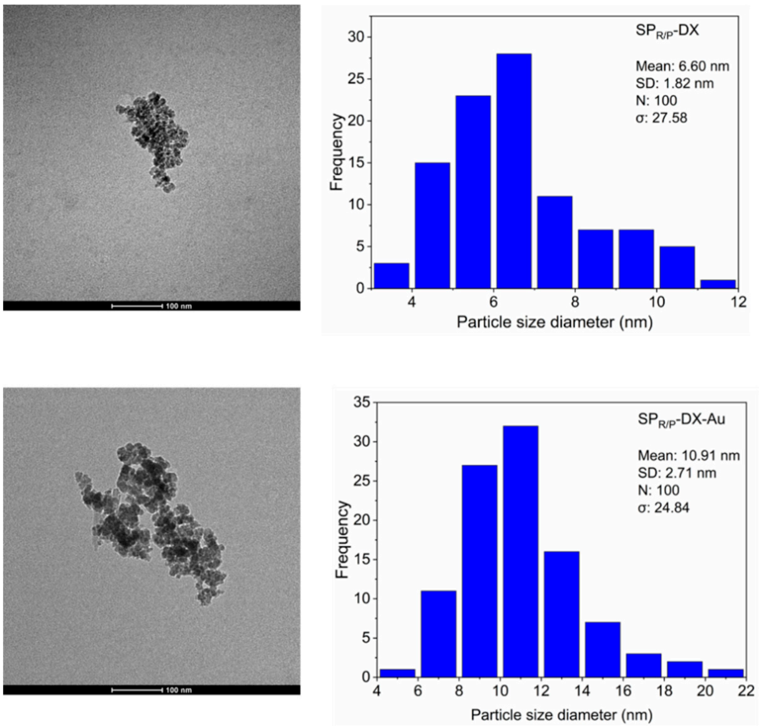

Figure S7. Transmission electron microscopy images of the SPIONs respective size histogram: Top: SP$_{R/P}$-Dx; bottom: SP$_{R/P}$-Dx-Au.

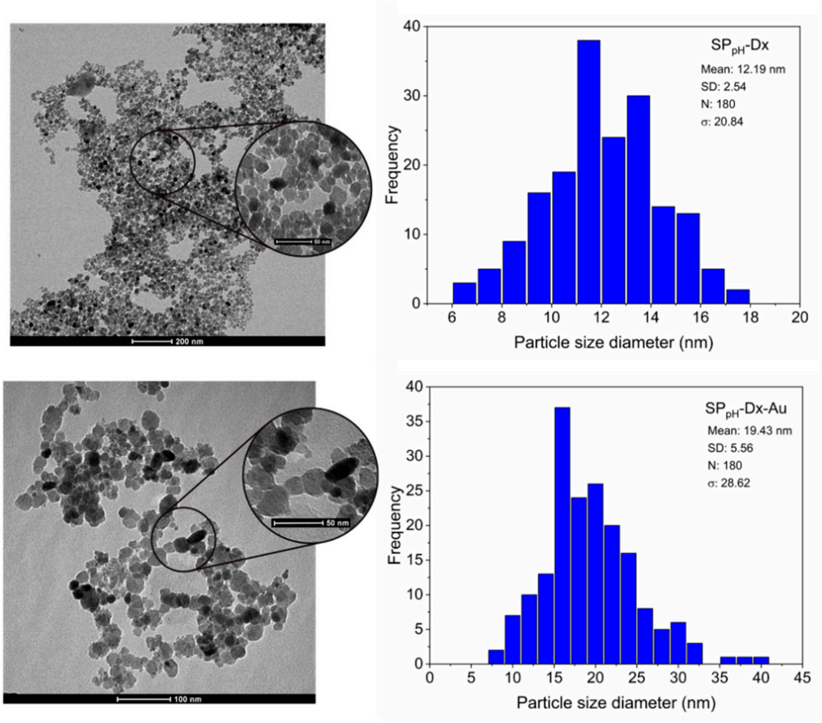

Figure S8. Transmission electron microscopy images of the SPIONs respective size histogram: Top: SP$_{pH}$-Dx; bottom: SP$_{pH}$-Dx-Au.

6. *Mössbauer Spectroscopy*

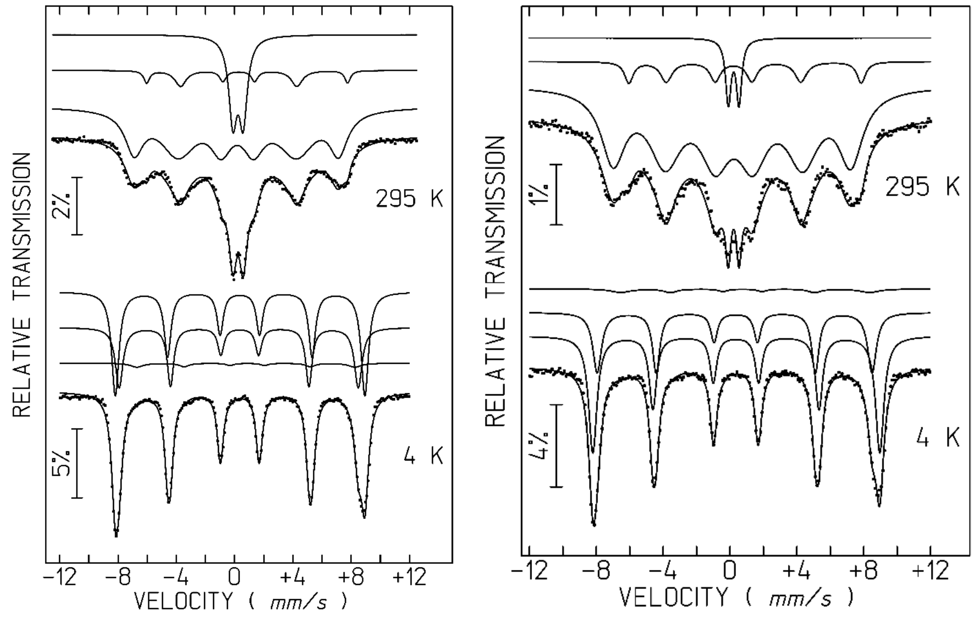

Figure S9. Mössbauer spectra of $SP_{R/P}$ (left) and $SP_{R/P}$-Dx-Au (right) taken at different temperatures. The lines over the experimental points are the calculated curves. The estimated parameters are collected in Table S3.

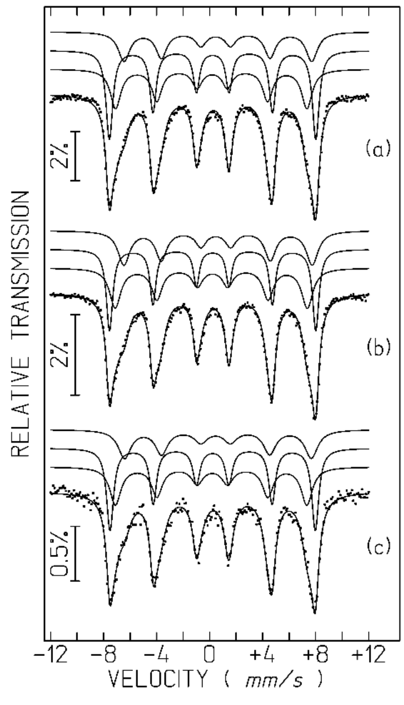

Figure S10. Room temperature Mössbauer spectra of (a) SP_{pH}-Dx (b) SP_{pH}-Dx-Au and (c) SP_{pH}-Dx-Au-Gd samples. Calculated lines on the experimental points are the sum of three sextets (see Table S3).

Table S4. Estimated parameters from the Mössbauer spectra of selected SPIONs samples at room temperature and at 4 K.

Sample	IS mm/s	ε mm/s	B_{hf} tesla	I (%)	Fe state	Fe in Fe_3O_4
**$SP_{R/P}$**	0.30	-0.12	43.5	76%	Fe^{3+} γFe_2O_3 , Fe_3O_4	**11%**
**295 K**	0.35	0.65	-	16%	Fe^{3+} in the smallest NPs	
	0.69	0.66	42.7	8%	$Fe^{2.5+}$ CN = 6 Fe_3O_4	
**4 K**	0.43	-0.07	50.9	39%	Fe^{3+} CN=4 γFe_2O_3 , Fe_3O_4	
	048	0.02	53.1	57%	Fe^{3+} CN = 6 γFe_2O_3 , Fe_3O_4	
	0.93	-0.10	46.4	3.8%	Fe^{2+} CN = 6 Fe_3O_4	
**$SP_{R/P}$-Dx**	0.29	-0.09	43.4	75%	Fe^{3+} γFe_2O_3 , Fe_3O_4	**11%**
**295 K**	0.35	0.69	-	17%	Fe^{3+} in the smallest NPs	
	0.68	0.63	42.1	8%	$Fe^{2.5+}$ CN = 6 Fe_3O_4	
**4 K**	0.42	-0.08	50.9	38%	Fe^{3+} CN=4 γFe_2O_3 , Fe_3O_4	
	048	0.01	53.0	58%	Fe^{3+} CN = 6 γFe_2O_3 , Fe_3O_4	
	0.93	-0.45	46.8	3.8%	Fe^{2+} CN = 6 Fe_3O_4	
**$SP_{R/P}$-Dx-Au**	0.29	-0.12	44.1	86%	Fe^{3+} γFe_2O_3 , Fe_3O_4	**13%**
**295 K**	0.33	0.64	-	5%	Fe^{3+} in the smallest NPs	
	0.67	0.69	43.1	9%	$Fe^{2.5+}$ CN = 6 Fe_3O_4	
**4 K**	0.43	-0.07	51.0	38%	Fe^{3+} CN=4 γFe_2O_3 , Fe_3O_4	
	048	0.02	53.3	58%	Fe^{3+} CN = 6 γFe_2O_3 , Fe_3O_4	
	0.94	0.23	46.5	4.4%	Fe^{2+} CN = 6 Fe_3O_4	
**SP_{pH}**	0.26	-0.09	44.3	32%	Fe^{3+} CN=4 γFe_2O_3 , Fe_3O_4	**33%**
**295 K**	0.33	0.01	48.6	47%	Fe^{3+} CN = 6 γFe_2O_3	
	0.66	0.26	42.8	21%	$Fe^{2.5+}$ CN = 6 Fe_3O_4	
**4 K**	0.43	0.00	51.6	34%	Fe^{3+} CN=4 γFe_2O_3 , Fe_3O_4	
	0.51	-0.03	53.6	56%	Fe^{3+} CN = 6 γFe_2O_3 , Fe_3O_4	
	0.94	-0.31	46.9	11%	Fe^{2+} CN = 6 Fe_3O_4	

**SP_{pH}-Dx**	0.27	-0.08	44.8	31	Fe^{3+} CN=4 γFe_2O_3 , Fe_3O_4	**39%**
**295 K**	0.34	-0.01	48.3	43	Fe^{3+} CN = 6 γFe_2O_3	
	0.65	0.17	43.9	26	$Fe^{2.5+}$ CN = 6 Fe_3O_4	
**SP_{pH}-Dx-Au**	0.28	-0.10	44.7	33	Fe^{3+} CN=4 γFe_2O_3 , Fe_3O_4	**45%**
**295 K**	0.34	-0.01	48.3	37	Fe^{3+} CN = 6 γFe_2O_3	
	0.66	0.17	44.1	30	$Fe^{2.5+}$ CN = 6 Fe_3O_4	
**SP_{pH}-Dx-Au-Gd**	0.27	-0.10	45.0	34	Fe^{3+} CN=4 γFe_2O_3 , Fe_3O_4	**44%**
**295 K**	0.34	0.00	48.1	37	Fe^{3+} CN = 6 γFe_2O_3	
	**0.65**	**0.19**	**43.9**	**29**	**$Fe^{2.5+}$ CN = 6 Fe_3O_4**	

IS isomer shift relative to metallic α-Fe at 298 K; $\varepsilon = (e^2QV_{zz}/4)(3\cos^2\theta - 1)$ quadrupole shift, B_{hf} magnetic hyperfine field. I relative area. CN coordination number. Estimated errors ≤ 0.02 mm/s for IS, ε, Γ, < 0.3 T for B_{hf} and < 2% for I.

7. *Magnetization Measurements*

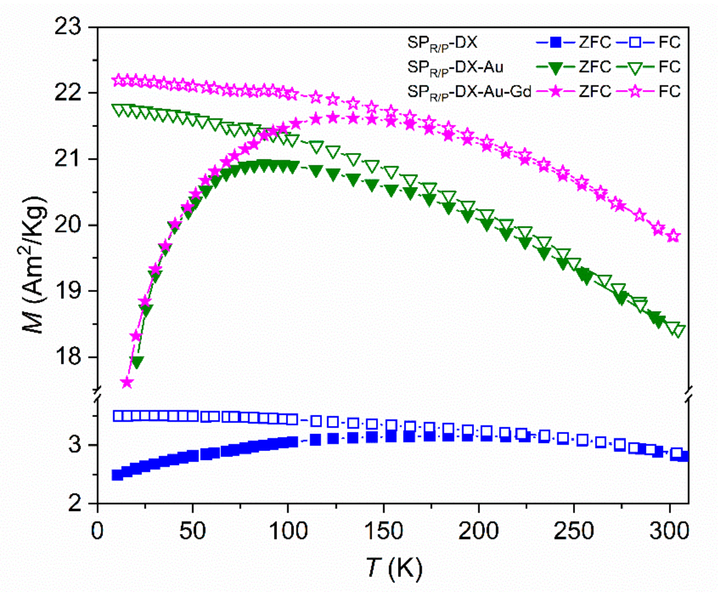

Figure S11. Temperature dependence of the zero-field cooling (ZFC) and field cooling (FC) magnetization for samples, SP$_{R/P}$-Dx, at 10mT (squares), SP$_{R/P}$-Dx-Au, at 50 mT (down triangles) and SP$_{R/P}$-Dx-Au-Gd, at 50 mT (stars).

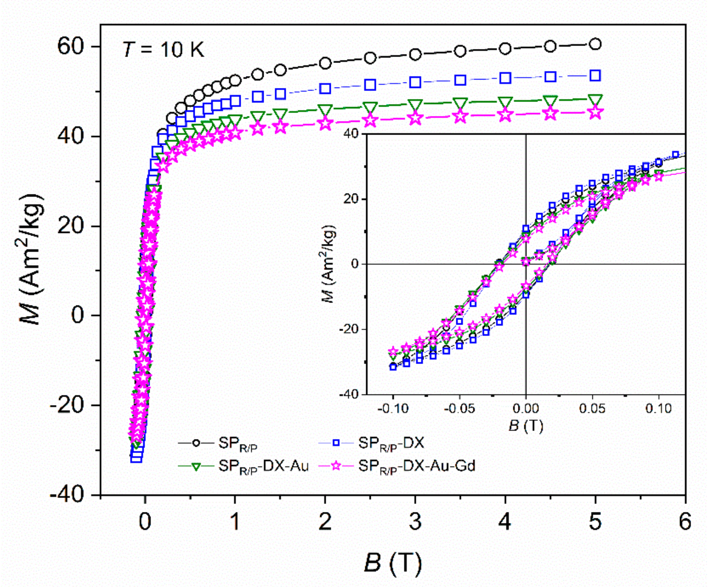

Figure S12. Magnetic field (*B*) dependence of magnetization (*M*) at 10 K for SP$_{R/P}$-based samples, SP$_{R/P}$ (circles), SP$_{R/P}$-Dx (squares), SP$_{R/P}$-Dx-Au (down triangles) and SP$_{R/P}$-Dx-Au-Gd (stars).

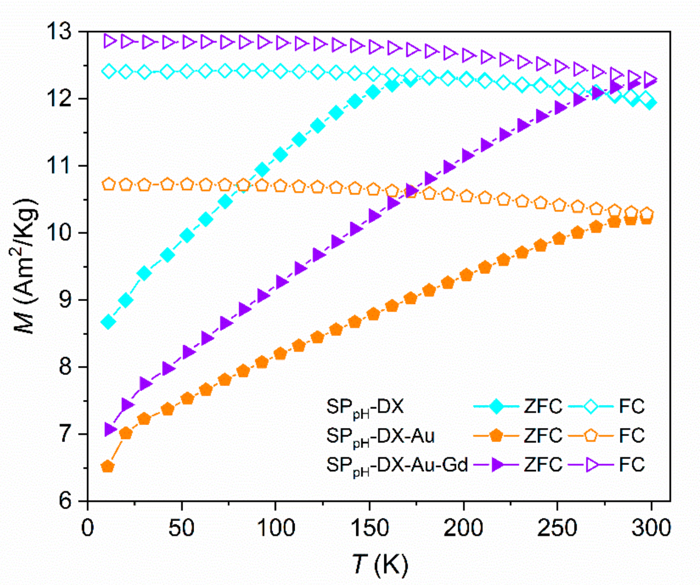

Figure S13. Temperature dependence of the zero-field cooling (ZFC) and field cooling (FC) magnetization (at 10 mT) for SP_{pH}-based samples, SP_{pH}-DX (diamonds), SP_{pH}-DX-Au (pentagons), and SP_{pH}-DX-Au-Gd (lying triangles).

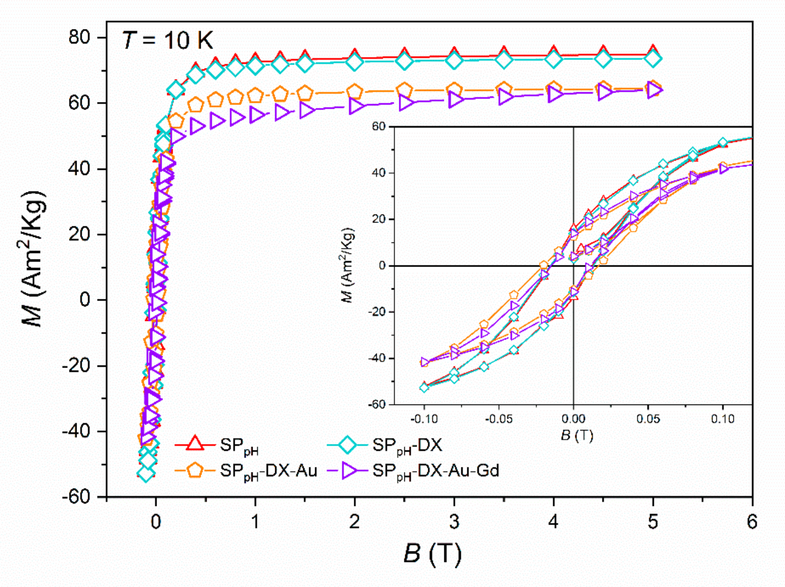

Figure S14. Magnetic field (B) dependence of magnetization (M) at 10 K for SP_{pH}-based samples, SP_{pH} (triangles) SP_{pH}-DX (diamonds), SP_{pH}-DX-Au (pentagons), and SP_{pH}-DX-Au-Gd (lying triangles).

8. *Relaxativity Studies*

Table S5. Longitudinal (r_1) and transverse (r_2) relaxivity values determined for selected samples at 300 MHz, 7 T, room temperature, Fe and Gd concentrations, and r_2/r_1 ratios.

Sample	7 T (300 MHz, room temp) / $mM^{-1}s^{-1}$			Concentration (measured by ICP) / M		Relaxivities considering Fe and Gd contributions, 7 T		
	r_1 (Gd)	r_2 (Fe)	r_2/r_1	[Fe]	[Gd]	r_1 (Gd + Fe)	r_2 (Fe + Gd)	r_2/r_1
$SP_{R/P}$	--	122	---	$1.2x10^{-3}$	---	0.4	122	340
$SP_{R/P}$-Dx-Au-Gd	5	10	2	$3.5x10^{-4}$	$3.3x10^{-5}$	0.4	9	23
SP_{pH}	--	186	---	$4.6x10^{-4}$	---	0.8	186	240
SP_{pH}-Dx-Au-Gd	55	56	1	$7.1x10^{-4}$	$2.6x10^{-6}$	0.2	56	282

Table S6. Longitudinal (r_1) and transverse (r_2) relaxivity values determined for selected samples at 300 MHz, 1.41 T, room temperature, Fe and Gd concentrations, and r_2/r_1 ratios.

Sample	1.41 T (60 MHz, 25°C) / $mM^{-1}s^{-1}$			Concentration (measured by ICP) / M		Relaxivities considering Fe and Gd contributions, 1.41 T		
	r_1 (Gd)	r_2 (Fe)	r_2/r_1	[Fe]	[Gd]	r_1 (Gd + Fe)	r_2 (Fe + Gd)	r_2/r_1
$SP_{R/P}$	---	61.8	---	$6.5x10^{-4}$	---	1.8	62.0	35
$SP_{R/P}$-Dx-Au-Gd	5.5	7.7	1.4	$2.7x10^{-4}$	$1.3x10^{-5}$	0.3	7.7	29
SP_{pH}	---	169.7	---	$1.6x10^{-4}$	---	7.7	169.7	22
SP_{pH}-Dx-Au-Gd	23.2	11.7	0.5	$5.3x10^{-4}$	$2.2x10^{-6}$	0.1	11.8	123
